# Shank2 identifies a subset of glycinergic neurons involved in altered nociception in an autism model

**DOI:** 10.1186/s13229-023-00552-7

**Published:** 2023-06-14

**Authors:** Florian Olde Heuvel, Najwa Ouali Alami, Oumayma Aousji, Esther Pogatzki-Zahn, Peter K. Zahn, Hanna Wilhelm, Dhruva Deshpande, Elmira Khatamsaz, Alberto Catanese, Sarah Woelfle, Michael Schön, Sanjay Jain, Stefanie Grabrucker, Albert C. Ludolph, Chiara Verpelli, Jens Michaelis, Tobias M. Boeckers, Francesco Roselli

**Affiliations:** 1 Department of Neurology, Ulm University, Ulm, Germany; 2 International PhD Program, Ulm University, Ulm, Germany; 3 Department of Anesthesiology, Intensive Care and Pain Medicine, University Hospital Münster, Münster, Germany; 4 Clinic for Anesthesiology, Intensive Care and Pain Medicine, University Hospital Bergmannsheil Bochum, Bochum, Germany; 5 Department of Biophysics, Ulm University, Ulm, Germany; 6 Institute of Anatomy and Cell Biology, Ulm University, Ulm, Germany; 7 Department of Internal Medicine (Renal), Pathology and Immunology, Washington University School of Medicine, St. Louis, USA; 8 German Center for Neurodegenerative Diseases (DZNE), Ulm, Germany; 9 Institute of Neuroscience, National Science Council, Milan, Italy; 10 Center for Biomedical Research (ZBF), Helmholtzstraße 8/2, 89081 Ulm, Germany; 11 Department of Anatomy and Cell Biology, Ulm University, Albert-Einstein Allee 11, 89081 Ulm, Germany

**Keywords:** Shank2, Autism spectrum disorder, Nociception, Spinal cord, Glycinergic interneurons

## Abstract

**Background:**

Autism Spectrum Disorders (ASD) patients experience disturbed nociception in the form of either hyposensitivity to pain or allodynia. A substantial amount of processing of somatosensory and nociceptive stimulus takes place in the dorsal spinal cord. However, many of these circuits are not very well understood in the context of nociceptive processing in ASD.

**Methods:**

We have used a Shank2^−/−^ mouse model, which displays a set of phenotypes reminiscent of ASD, and performed behavioural and microscopic analysis to investigate the role of dorsal horn circuitry in nociceptive processing of ASD.

**Results:**

We determined that Shank2^−/−^ mice display increased sensitivity to formalin pain and thermal preference, but a sensory specific mechanical allodynia. We demonstrate that high levels of Shank2 expression identifies a subpopulation of neurons in murine and human dorsal spinal cord, composed mainly by glycinergic interneurons and that loss of Shank2 causes the decrease in NMDAR in excitatory synapses on these inhibitory interneurons. In fact, in the subacute phase of the formalin test, glycinergic interneurons are strongly activated in wild type (WT) mice but not in Shank2^−/−^ mice. Consequently, nociception projection neurons in laminae I are activated in larger numbers in Shank2^−/−^ mice.

**Limitations:**

Our investigation is limited to male mice, in agreement with the higher representation of ASD in males; therefore, caution should be applied to extrapolate the findings to females. Furthermore, ASD is characterized by extensive genetic diversity and therefore the findings related to Shank2 mutant mice may not necessarily apply to patients with different gene mutations. Since nociceptive phenotypes in ASD range between hyper- and hypo-sensitivity, diverse mutations may affect the circuit in opposite ways.

**Conclusion:**

Our findings prove that Shank2 expression identifies a new subset of inhibitory interneurons involved in reducing the transmission of nociceptive stimuli and whose unchecked activation is associated with pain hypersensitivity. We provide evidence that dysfunction in spinal cord pain processing may contribute to the nociceptive phenotypes in ASD.

**Supplementary Information:**

The online version contains supplementary material available at 10.1186/s13229-023-00552-7.

## Background

Autism Spectrum Disorders (ASD) are characterized by a range of sensory abnormalities, in particular in the domain of tactile sensitivity [1]. Among these, abnormal nociception is strikingly common in ASD and it manifests itself either as hypo-sensitivity or as hyper-sensitivity to painful stimuli. The common occurrence of self-injury, self-mutilation (including cases of self-extraction of teeth) and unreported wounds [2], supported by clinical and experimental studies [3–5] has been interpreted as evidence of reduced sensitivity to painful stimuli in ASD patients. Conversely, a subset of ASD patients display hyperalgesia and pain hypersensitivity in the form of mechanical/tactile allodynia [6], static mechanical allodynia (pain in response to light touch/pressure [7, 8], dynamic mechanical allodynia (pain in response to stroking lightly), reduced threshold for thermal pain [9], movement allodynia (pain triggered by normal movement of joints or muscles) and chronic pain unrelated to medical conditions [10–12]. Pain hypersensitivity may constitute a major and underappreciated source of discomfort for ASD patients, in particular due to their inefficient communication capabilities that afflict most of the affected ASD patients [2], resulting in unnecessary medical procedures [10].

It is worth noting that ASD mouse models, carrying mutations in different genes, may display hyper- or hyposensitivity to pain, suggesting that the variability of the clinical phenotype may be linked to the genetic heterogeneity of ASD (more than 800 genes are linked to ASD [13]). In fact, each genetic mutation might disrupt a discrete but different node of the nociceptive circuit, leading to mutation-specific phenotypes and mechanisms. In particular, loss of the ASD-related Shank3 protein results in pain hyposensitivity due to the disruption of the scaffold architecture, enabling TRPV1 signaling in dorsal root ganglion (DRG) neurons [14].

Since disturbed nociception is a major source of discomfort for patients, knowledge of the involved neuronal circuits may provide insights into diagnosis and treatment. Although substantial processing of somatosensory and nociceptive stimuli takes place in circuits in the dorsal spinal cord [15], we largely ignore the extent of the involvement of these circuits in ASD-associated nociceptive phenotypes, either in terms of the cellular subpopulations involved or in terms of molecular and neurochemical abnormalities at work. One could speculate that, since a large fraction of ASD-associated genes code for synaptic proteins [16] change in the synaptic architecture, connectivity and excitation/inhibition balance may be altered in the spinal cord of ASD patients and murine models.

Here we consider the ASD model obtained by deleting the gene coding for the postsynaptic density (PSD)-enriched scaffold protein Shank2 [17]. Indeed, point mutations and missense mutations in Shank2 are responsible for a small but consistent fraction of ASD cases [18].

Shank2^−/−^ mice (obtained by targeting exon-6 and exon-7; [19, 20]), are considered a *bona fide* mouse model of ASD. In fact, they display a phenotype characterized by reduced social interaction, increased anxiety and compulsive grooming. Recent work has suggested that Shank2^−/−^ mice may display abnormal nociception due to alteration of spinal cord circuits [21], although it must be stressed that deletion of different exons in distinct Shank2^−/−^ models may also lead to different phenotypes [22]. Nevertheless, the neuronal subpopulations and the circuit architectural features responsible for such phenotype remain to be investigated.

Here we show that Shank2^−/−^ mice display hypersensitivity to formalin-induced pain; notably, we have identified (in murine and human spinal cord) a subpopulation of glycinergic interneurons characterized by very high expression levels of Shank2. Loss of Shank2 results in the reduction in synaptic NMDAR and in the blunted recruitment of these inhibitory interneurons upon painful stimuli, which results in the over-activation of lamina-I nociceptive projection neurons.

## Materials and methods

### Animals

All experiments were performed in agreement with the local and national guidelines for animal experimentation. In particular, all experiments were approved by the Regierungspräsidium Tübingen under the licence n° o.103 and by the Italian Ministry of Health 966/2016-PR.

All transgenic mouse lines used were previously described. Glycine transporter 2 (GlyT2)-EGFP mice [23] tissue samples were a courtesy of Silvia Arber; spinal cord samples from parvalbumin (PV)-Cre x tdTomato-ROSA26 [24] were a courtesy of Pico Caroni. Vesicular GABA transporter (VGAT)-Cre x tdTomato-ROSA26 [25], pancreas associated transcription factor 1a (Ptf1a)-Cre x tdTomato-ROSA26 [26], paired related homeobox protein-like 1 (Prrxl1)-Cre x tdTomato-ROSA26 (also known as DRG11); [27] and vesicular glutamate transporter 2 (vGluT2)-CRE x tdTomato-ROSA26 [25] double transgenic mice were a courtesy of Filippo Rijli, Ahmad Bechara and Alberto Loche. Ret-GFP transgenic mice were previously reported [28]. Shank2^−/−^(Δ7) mice were previously described [19].

### Shank2 antibodies

Polyclonal rabbit antiserum against the C-terminus of Shank2 (SA5192), custom made, was previously described [29]. Briefly, partial cDNAs of the ProSAP1 cDNA (encoding amino acids 826–1259) were subcloned into the bacterial expression vector pGEX-1T (Pharmacia Biotech, Uppsala, Sweden). A 95 kDa glutathioneS-transferase (GST)-ProSAP1 fusion protein was expressed in Escherichia coli XL 1 Blue and used to immunize rabbits.

### pEGFP–ProSAP1 construct

pEGFP–ProSAP1 constructs were provided by Prof Boeckers and was previously reported [30]. Full-length ProSAP1 cDNAs were cloned into the pEGFP (C1-3) vector (Clontech, Palo Alto, CA, USA) coding for fusion proteins with the GFP at the N-terminus.

### HEK cell culture and Shank2 transfection and overexpression

HEK cells (Human embryonic kidney 293 cells) were cultured in 6-well plates in DMEM (10% FCS and 1% Pen/strep) with a density of 80.000 cells per well. 1 day after plating, cells were transfected with 1 µg of shank2 plasmid (pEGFP-ProSAP1) with PEI protocol [31] overnight, followed by change of medium, washing and harvesting of cells. Extraction of protein was performed as described below [32, 33].

### Western blot

Western blot for Shank2 was performed as previously reported [32]. Briefly, the lumbar spinal cord, cortex, cerebellum, hippocampus, striatum, and dorsal root ganglia were dissected from wild type (WT) and Shank2^−/−^ mice (sacrificed by cervical dislocation). Tissue samples were snap-frozen on dry ice and quickly homogenized in complete radioimmunoprecipitation assay (RIPA) buffer with Protease and Phosphatase inhibitors using a mortar. The homogenate was subject to sonication and then cleared by centrifugation (10,000 g, 10 min, 4 °C). Protein concentration was measured by BCA kit and 20 µg of protein were loaded in each lane of an 8%-acrylamide gel. As a positive control, protein extract (2 µL) from an overexpression of Shank2, using a plasmid (pEGFP-ProSAP1), in HEK cells was used. As negative control proteins from untransfected HEK cells were used. Proteins were transferred to a nitrocellulose membrane using a Trans-Blot® Turbo™ Transfer System (Bio-Rad) semi-dry transfer apparatus (standard protocol for mixed molecular weight was run twice); membranes were blocked in 5% bovine serum albumin (BSA) in tris buffered saline (TBS) added with 1% Tween20 for 1 h and incubated with anti-Shank2 SA5192 antibody (diluted 1:500 in blocking solution) overnight at 4 °C on an orbital shaker. After 6 washing steps of 20 min with TBS added with 0.25% Tween the membranes were incubated with a horseradish peroxidase (HRP) coupled secondary antibody from Bio-Rad® for 1 h at room temperature, diluted in blocking solution (5% BSA in TBS added with 1% Tween20). The HRP coupled blots were then incubated in Clarity Western enhanced chemiluminescent (ECL) HRP substrate solution (Bio-Rad) for 5 min. Detection was performed with a digital CCD-camera based system from Fuji (Tokio, Japan). Membranes were then re-incubated at room temperature for 1 h with anti-ꞵ-tubulin antibody (1:1000 in blocking buffer). Quantification was performed with imageJ Fiji toolbox, normalizing each band on ꞵ-tubulin.

### Intraspinal injection of AAV9-GFP for sparse labelling

Intraspinal injection of adeno associated virus (AAV9) was performed as previously reported [34]. Briefly, WT mice aged P20 were administered buprenorphine (0.05 mg/Kg) and meloxicam (0.1 mg/Kg) 30 min before being anesthetized with sevoflurane (4% in 96% O_2_, 800 ml/min). Dorsal skin was shaved and incised (10 mm) at lumbar level. Paraspinal muscles were blunt-dissected and dorsal laminae were sectioned at vertebrae T11-T13 level. Upon removal of the dorsal laminae bone flap, the underlying dura was opened using a 33G needle and washed with artificial cerebrospinal fluid (ACSF). Viral injections were performed at the coordinates (y =  + 0.22–25 mm; z =  − 0.55 to 6 mm) having the central dorsal artery as reference. A total of four injections were performed, 250 nl/5 min each, using a pulled-glass capillary connected to a Picospritzer-III apparatus. Muscles were thereafter sutured on the midline using Prolene 7.0 surgical thread; the fascia was repositioned to cover the wound, and the skin was stiched on the midline using Prolene 6.0 surgical thread. Mice were then transferred to a warmed recovery cage with facilitated access to food and water and were administered additional doses of buprenorphine every 12 h for the following 72 h.

### CTB retrograde labeling

Retrograde labeling of sensory neurons and their central afferents in the dorsal spinal horn was performed as previously reported [35]. Briefly, adult WT mice were anesthetized using isoflurane, and the injection site was shaved. 0.1–0.3 µl of Choleratoxin subunit B (CTB) conjugated with Alexa Fluor 488 (2 µg/µl in phosphate buffered saline (PBS); Thermofischer) was injected into the hairy skin of leg using a fine glass capillary. Mice were sacrificed 7 days after the injection and further processed.

### Immunostaining

Spinal cord samples were processed as previously reported [36]. Briefly, mice were perfused with 4% paraformaldehyde (PFA) in PBS, (L1–L5) lumbar spinal cord was isolated and post-fixed for 18 h in 4% PFA. Tissue samples were thereafter washed and cryoprotected overnight at 4 °C in 30% sucrose. After embedding in optimal cutting temperature (OCT, Tissue-Tek), 40 µm cryostat sections were obtained. Subsequently sections were blocked, incubated for 48-72 h with primary antibodies (see Additional file 1: Table S1) diluted in PBS with 3% BSA, 0.3% Triton X-100 at 4 °C on an orbital shaker. Sections were then washed with PBS (30 × 3 min at RT) and incubated for 2 h at room temperature (RT) with appropriate combinations of secondary antibodies (see Additional file 1: Table S1) and DAPI (1:1000) for nuclear staining. For Isolectin GS-IB_4_ staining, a directly Alexa Fluor 488-conjugated antibody (Invitrogen, cat. 121411) was used to label nonpeptidergic nociceptive primary afferents in *laminae* II, at the concentration of 1:1000 diluted in 10% normal donkey serum (NDS) additioned with 0.3% Triton-x. After additional washing in PBS (3 × 30 min at RT), the sections were dried and mounted with Prolong antifade mounting medium (Invitrogen). For stimulated emission depletion (STED) Microscopy, before imaging, the samples were washed shortly with dH_2_0 and mounted in 2,2-thiodiethanol (TDE) buffer.

### Clarity and immunostaining on human spinal cord samples

Human spinal cord samples were obtained in agreement with the procedures approved by the ethical committee of Ulm University (n.245/17), full data on human samples is available in Additional file 1: Table S2. Clearing and immunostaining were performed on 100 µm thick post-mortem human lumbar spinal cord sections as previously described [37]. Briefly, sections were incubated in hydrogel solution (40% acrylamide, 4% PFA, 0.25% VA-044 initiator; pH = 7.3) at 4 °C for 1 week. Sections were then degassed using a desiccator and polymerized at 37 °C for 1.5 h until the hydrogel has completely hardened. The excess of hydrogel was afterward removed by washing for 1.5 h at 37 °C with PBS. The samples were then passively cleared in a clearing solution (4% SDS, 200 mM boric acid solution; pH = 7.3) for 1 week at 37 °C. The clearing solution was afterward washed away for 1 day at 37 °C with 0.1% TritonX-100. Once cleared, the sections were stained starting with a blocking step in 3% BSA and 0.3% TritonX-100 for 2 h at RT. After blocking, sections were incubated in primary antibody (Additional file 1: Table S1) for 72 h at 4 °C followed by a washing step in 0.1% TritonX-100 3 times for 30 min. Subsequently, sections were incubated in secondary antibodies for 2 h at RT, and washed 3 times for 30 min in 0.1% TritonX-100. Sections were finally mounted on microscope glass slides with Prolong antifade mounting medium (Invitrogen).

Overview images of the spinal cord sections were taken with a fluorescent microscope (Keyence, BZ-X800) equipped with a 4 × air objective. Higher resolution images were then performed using a laser-scanning confocal microscope (Zeiss LSM 980, Carl Zeiss), with a 20 × air or 100 × oil objective, by taking tile-scan images of the grey matter or single neurons in laminae I–IV.

### In situ single-mRNA hybridization

Fluorescence in situ single mRNA hybridization [38] was performed according to manufacturer instructions (Fluorescence In Situ mRNA Hybridisation for fixed frozen tissue, RNAscope by ACDBio) with small modifications (as previously reported; [39]). Shortly, sections were mounted and dried on glass slides, quenching of autofluorescence was performed by pretreatment with 0.1 M Glycine in PBS for 15 min. Thereafter, a 3 min antigen retrieval step was performed, and sections were washed twice in dH_2_O and once in ethanol. Then pretreatment reagent III (all reagents were provided by ACDbio) was applied for 20 min at 40 °C. The Probes (GlyT2 and c-FOS) were hybridized for 4.5 h at 40 °C, followed by a wash step of 2 × 2 min at RT. The sections were then incubated for 30 min with amplification-1 reagent at 40 °C, followed by a wash step of 2 × 2 min at RT. Subsequently, the sections were incubated for 15 min with amplification-II reagent at 40 °C, and washed 2 × 2 min at RT. The last amplification step was performed by incubating the sections for 30 min with amplification-III reagent at 40 °C, followed by a wash step of 2 × 2 min at RT. After this, the sections were incubated with amplification-IV for 45 min at 40 °C, followed by a final wash step of 2 × 10 min at RT. Sections were counterstained with DAPI or co-immunostained with specific markers of interest. For the co-immunostaining, the sections were blocked (3% BSA, 0.3% Triton in PBS) for 1 h, then incubated overnight at 4 °C with primary antibodies, diluted in blocking buffer (see Additional file 1: Table S1). After the incubation the sections were washed for 3 × 30 min in 1X PBS. Subsequently, a 2 h incubation with secondary antibody diluted in the blocking buffer at RT (Donkey anti-guinea pig 568, 1:500 [invitrogen]) was performed. After the last washing steps (3 × 30 min in PBS), the sections were counterstained with DAPI and mounted using Fluorogold prolong antifade mounting medium (Invitrogen).

### Stimulated emission depletion microscopy (STED)

A custom build dual-color setup, with a high NA objective (HCX PL APO 100x/1.40–0.70 oil CS, Leica), was used for STED microscopy as described previously [40]. Briefly, a supercontinuum laser source (SC-450-PP-HE, Fianium) with a broad spectral range provided all the excitation (568 nm and 633 nm) and depletion (~ 720 nm and ~ 750 nm) beams, samples were scanned with a piezo stage (733.2DD, Physik Instrumente) and emission was recorded by an avalanche photodiode (SPCMAQRH-13/14-FC, Perkin- Elmer). Acquisition mode could be switched between confocal (diffraction limited resolution) or STED (lateral resolution ~ 35 nm) and was controlled by a custom LabVIEW (National Instruments) software.

### Confocal imaging

Confocal images were acquired using a LSM-710 or LSM-980 (Carl Zeiss AG) microscope as previously reported [34], fitted with a 20× air objective or with 40×, 60× or 100× oil objectives with optical thickness fitted to the optimum value. For overview images, 20× objective 6 × 6 image tiles were acquired. For high-magnification images, a zoom factor 3× was applied during the acquisition of 60× or 100× oil objective images, oversampled in the z-axis to twice the theoretical optimal value. All images have been acquired at 1024 × 1024 pixels resolution and 12- or 16-bit depth. Acquisition parameters were set to avoid over or under-saturation and kept constant for each experimental set.

### Image analysis

For image analysis, confocal pictures were loaded into ImageJ. For the definition of Shank2 immunostaining intensity, a circular region of interest was manually located around each neuronal nucleus (identified in NeuN staining) and the integrated fluorescence intensity (expressed in arbitrary units -au- ranging between 0 and 4095) averaged over the area of the region of interest, was logged. For the identification of Shank2^high^ neurons, we defined a threshold by considering the top of the distribution of intensities in laminae I and II and adding 200 au, thus defining as Shank2^high^ any neuron whose average fluorescence was at least 200 au higher than the brightest neuron in laminae I and II. Operationally, Shank2 immunofluorescence images were thresholded until neurons in laminae I and II were no longer visible and the threshold value was then moved 200 au higher; any Shank2 + neuron still visible at this stage was considered Shank2^high^. In the quantification of Shank2 expression in human samples, the contour of neurons was manually drawn using the SMI-311 immunostaining as reference and the average Shank2 immunostaining intensity was quantified. For the analysis of Homer, GluN1, vGluT1 and vGluT2 cluster size on GlyT2 + interneurons, the contour of each interneuron was manually drawn in imageJ; after thresholding, all clusters juxtaposed to the contour were highlighted and the size logged. The analysis of the c-Fos mRNA was performed by counting the single c-Fos mRNA dots per GlyT2 + interneuron (depicted with GlyT2 mRNA). Since c-Fos was expressed in every GlyT2 + neuron, we defined a threshold of at least 50 mRNA molecules per cell for it to be considered c-Fos positive. The neuronal and synaptic architecture was analysed by taking a region of interest in the predefined laminae and counting manually the number of neurons and with the Imaris software the density of synapses in the region of interest.

### Formalin test

Formalin test was performed according to the previously reported protocols [41, 42]. Briefly, animals were injected with 20 µl 1% formalin solution in the dorsal surface of the left hindpaw; mice were thereafter swiftly put in a plexiglas chamber and video recording was obtained for 60 min and scored off-line for the duration of biting, licking or flinching activities. Phase I (0–10 min) and phase II (30–40 min) composite scores were computed.

### Adhesive removal test

The adhesive removal test was performed as previously described [43]. Briefly, a small piece of adhesive tape was placed on the plantar of the left hind paw of each mouse. Mice were left to run free in the cage for 30 min, after each mouse the cage was cleaned with diluted alcohol. Nociceptive phenotype and general activity was measured over the complete time course by an animal tracking software (Ethovision XT; Noldus).

### Texture discrimination test

The texture discrimination test was performed to distinguish sensitive differences in sensory perception of the mice [44]. The cage was divided in two parts, one side with rough sandpaper (60 grit coarse) and the other with the smooth side of the same paper; to assure that the olfactory cues of the paper were identical throughout the box. The mice were allowed to freely explore the cage for 30 min. Between the animals, the pieces of sandpaper were changed, and the box was cleaned. Nociceptive phenotype, preference and general activity was measured over the complete time course by an animal tracking software (Ethovision XT; Noldus).

### Thermal place preference test

The thermal place preference test was performed to identify thermal allodynia and preference of the mice [45, 46]. The cage was divided in two parts, one side was set at an ambient room temperature (25 °C) and, in different experiments, the other side was set to either warm (45 °C) or cold (10 °C). The mice were allowed to freely explore the cage for 8 min. Between the animals, the box was cleaned. Nociceptive phenotype, preference and general activity was measured over the complete time course by an animal tracking software (Ethovision XT; Noldus).

### Hargreaves test after CFA-induced sensitization

Animals were injected with Complete Freund Adjuvant (CFA; 100 µl, Sigma) subcutaneously into the left hindpaw, as described previously [42]. CFA injection led to an obvious tissue inflammation of the hindpaw characterised by erythema, oedema, and hyperpathia. For testing, each animal was placed in a box containing a smooth, temperature-controlled glass floor. The heat source was focused on a portion of the hindpaw, which was flush against the glass, and a radiant thermal stimulus was delivered to that site. The stimulus was shut off when the hindpaw moved, or after 20 s to prevent tissue damage. The time from the onset of radiant heat to the endpoint was the paw withdrawal latency (PWL). Thermal stimuli were delivered 3 times to each hindpaw at 5–6 min intervals.

### Von frey test after CFA-induced sensitization

After CFA injection and induction of inflammation, von Frey filaments (VFFs) were employed to measure mechanical hyperalgesia according to established protocols [47]. Briefly, a series of calibrated VFFs (0.4–25.0 g) were applied perpendicularly to the plantar surface of the hindpaw with sufficient force to bend filaments for 60 s or until it withdrew. In the absence of a response, filament of next greater force was applied. In the presence of a response, filament of next lower force was applied. To avoid injury during experiments, cutoff strength of VFF was 25.0 g. The tactile stimulus producing a 50% likelihood of withdrawal was determined by means of the “up-down” calculating method, as described in detail previously [47]. Each test was repeated 2–3 times at ~ 2 min interval, and the average value was used as the force to induce a withdrawal response.

### Statistical analysis

Statistical analysis was performed with the Graphpad Prism version 8.00 for Windows (GraphPad Software, La Jolla California USA) software package. Group comparisons were performed using the Kruskal–Wallis or ANOVA test, with Dunnett's or Bonferroni's correction for multiple comparisons. When appropriate, an unpaired t-test was used. All results are depicted as histograms with average ± SD, scatterplots or box and whiskers (10–90 percentile) unless indicated. Statistical significance was set at *p* < 0.05.

## Results

### Shank2^−/−^ mice display hypersensitivity to inflammatory pain and sensory modality-specific allodynia

**Fig. 1:**
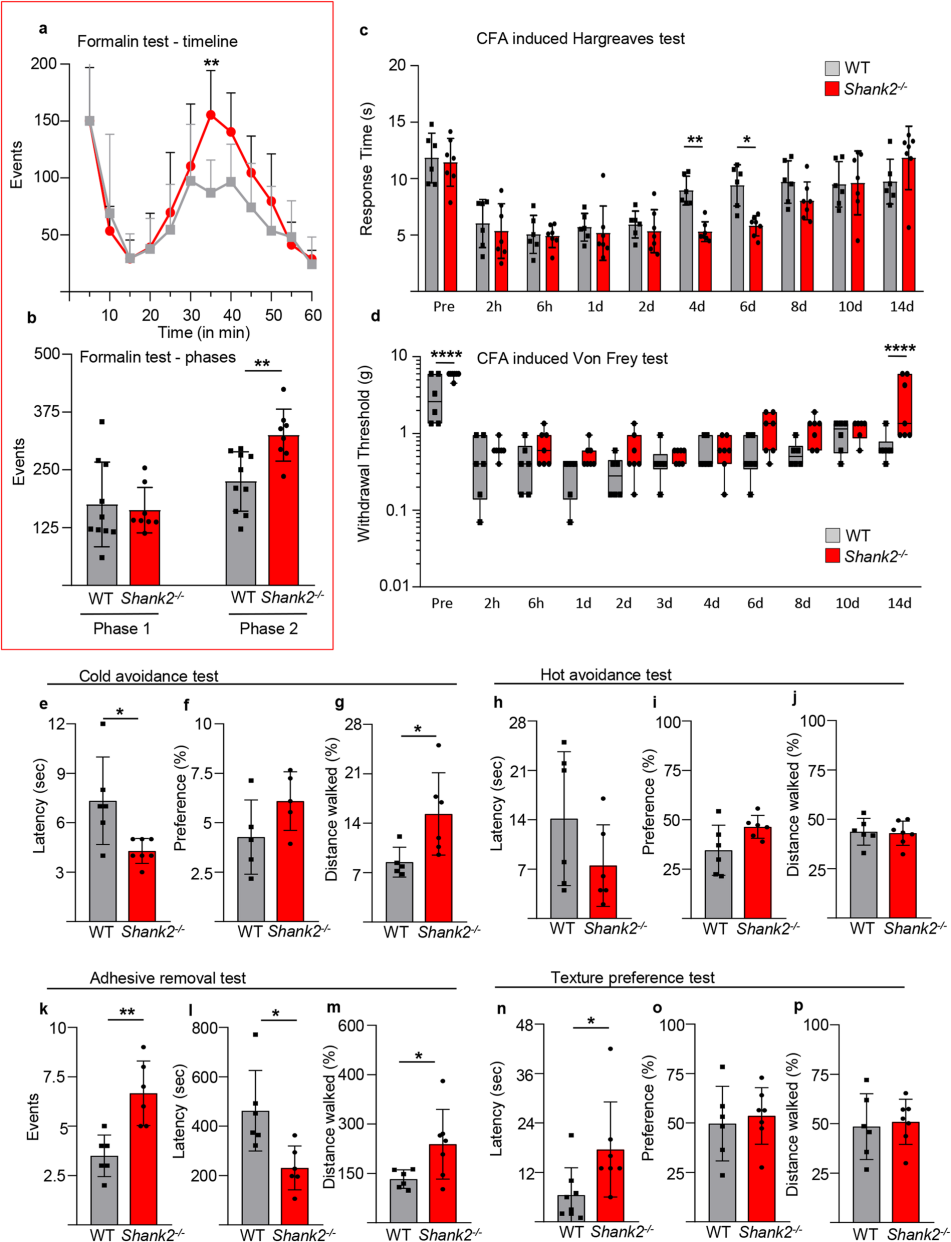
Shank2^−/−^ mice display hypersensitivity to inflammatory pain and sensory modality-specific allodynia. Behavioural analysis of nociceptive responses in Shank2^−/−^ and WT mice. **a** and **b** Formalin was injected in the hindpaw to measure the licking/flinching response. Shank2^−/−^ mice show no difference in licking/flinching in the first phase compared to WT (*p* > 0.05) after the formalin test. However, in the second phase (30–40 min) a significant increase was observed in licking/flinching in the Shank2^−/−^ mice (*p* < 0.01) (N = 10 for WT and N = 8 for Shank2^−/−^). **c** and **d** CFA induced thermal and mechanical hyperalgesia was measured. Thermal hyperalgesia was found in both WT and *Shank2*^−/−^ mice the first few days (1–4 days), normalisation of withdrawal time occured in the WT mice from 4 days, but a significant increased sensitivity remained in *Shank2*^−/−^ mice until day 6 (*p* < 0.05). Both WT and *Shank2*^−/−^ mice show an increased sensitivity in the first few hours and days of the von Frey filaments. No difference was observed between WT and *Shank2*^−/−^ mice in the first days following CFA injection. At day 14 *Shank2*^−/−^ mice show a decreased sensitivity compared to WT mice (*p* < 0.0001) (N = 6 for WT and N = 7 for *Shank2*^−/−^). **e**–**g** Latency to escape, preference and distance walked was measured at the thermal preference test (cold plate). Shank2^−/−^ mice displayed a significant decrease in latency on the cold plate compared to WT mice (*p* < 0.05), a comparable preference was shown between Shank2^−/−^ and WT (approx. 5% for both genotypes) and a significant increase of distance walked on the cold side (normalised to the distance walked on RT side) was seen in Shank2^−/−^ mice compared to WT mice (*p* < 0.05); (N = 6 for WT and N = 7 for Shank2^−/−^). **h**–**j** Latency to escape, preference and distance walked was measured in the thermal preference task (hot plate) between WT and Shank2^−/−^ mice. Shank2^−/−^ mice displayed no significant difference in latency compared to WT mice (*p* > 0.05), no difference in preference has been observed (approx. 40% for both genotypes) and no difference in distance walked (approx 40% for both genotypes); (N = 6 for WT and N = 7 for Shank2^−/−^). **k**–**m** An adhesive tape was stuck to the hindpaw of the mouse to measure tactile allodynia. Shank2^−/−^ mice showed an increase in removal attempts, latency to notice the adhesive tape and distance walked compared to WT mice (*p* < 0.01; *p* < 0.05 and *p* < 0.05 respectively) (N = 6 for WT and N = 7 for Shank2^−/−^). **n**–**p** Latency to escape, preference and distance walked has been measured in the texture discrimination task (sandpaper, 60 grit coarse). Shank2^−/−^ mice showed a significant increase in latency compared to WT mice (*p* > 0.05) and a comparable preference and distance walked was shown between Shank2^−/−^ and WT (approx. 50% for both genotypes) (N = 8 for WT and N = 7 for Shank2.^−/−^). Data shown as average ± SD. *p < 0.05; **p < 0.01

First, we verified if Shank2^−/−^ mice displayed abnormal nociceptive responses and pain processing using acute and injury-associated painful stimuli. We tested sensitivity to acute chemical/inflammatory nociception using the formalin test. The first phase of the formalin pain response test (between 0 and 10 min), reveal that the measure of nociceptors activation and transmission was unaffected by Shank2 deletion: the cumulative time spent by Shank2^−/−^ mouse licking or flinching the injected hindpaw was comparable with the one spent by WT littermates (WT vs Shank2^−/−^; 175 ± 91 vs 163 ± 49; Two-way Repeated Measures (RM) ANOVA; df = 32; *p* > 0.05; Fig. 1a, b). Conversely, licking/flinching time spent in the phase II, related to short term spinal plasticity, was significantly longer in Shank2^−/−^ mice (WT vs. Shank2^−/−^; 2255 ± 64 vs. 354 ± 56; Two-way RM ANOVA; *df* = 32; *p* < 0.01; Fig. 1a, b), indicating an increased pain perception [42, 48].

We further tested thermal and mechanical allodynia after chronic induction of inflammation by intraplantar injection of complete Freund’s adjuvant (CFA; [42]). After CFA injection, both WT and Shank2^−/−^ mice displayed a comparable decrease in the latency of paw withdrawal from a thermal source, indicating the appearance of inflammation-induced thermal hyperalgesia; however, starting from day 4, WT mice displayed a rapidly progressive normalization of the withdraw latency, whereas Shank2^−/−^ mice continued to manifest an increased sensitivity to thermal stimuli up to 6 days after CFA injection (WT vs. Shank2^−/−^ day 4: 8.9 ± 1.3 s vs. 5.3 ± 0.9 s; *p* < 0.01; day 6: 9.4 ± 1.8 s vs. 5.8 ± 0.9 s; *p* < 0.05; Two-way RM ANOVA; *df* = 9; Fig. 1c). Notably, thermal sensitivity before CFA administration and in the early phases of the inflammatory response was comparable in Shank2^−/−^ and WT mice. Thus, only the recovery of the inflammation-induced thermal hyperalgesia was affected by the Shank2 deletion.

Conversely, baseline sensitivity to mechanical stimulation by von Frei’s filaments was decreased in Shank2^−/−^ compared to WT mice (WT vs. Shank2^−/−^ pre: 3.3 ± 2.2 g vs. 5.8 ± 0.6 g; *p* < 0.0001; Two-way RM ANOVA; *df* = 10; Fig. 1d). After CFA injection, in contrast to thermal hyperalgesia and to the response to formalin injection, Shank2^−/−^ mice did not display an increased sensitivity to mechanical stimulation and actually displayed a trend towards faster resolution of the inflammation-induced mechanical allodynia, with a significant decreased sensitivity at day 14 (thus, decreased mechanical allodynia; WT vs. Shank2^−/−^ day 14: 0.7 ± 0.3 g vs. 2.9 ± 2.4 g; *p* < 0.0001; Two-way RM ANOVA; *df* = 10; Fig. 1d).

Since we had identified a hypersensitivity of Shank2^−/−^ to painful stimuli under sensitized conditions (formalin and CFA injection), we set out to further investigate if sensory or nociceptive phenotypes could be detected at baseline. We therefore exploited thermal preference (hot-plate and cold-plate) tests to investigate thermal allodynia, and adhesive tape test [43] and the sandpaper test [44] to investigate tactile allodynia.

In the thermal preference test-cold (10 °C; [45]) Shank2^−/−^ mice showed a decreased latency to escape the cold side compared to WT mice (WT vs Shank2^−/−^; 7.3 ± 2.7 s vs. 4.3 ± 0.8 s; unpaired *t*-test; *t* = 2.739; *df* = 11; *p* < 0.05; Fig. 1e); after the escape, both WT and Shank2^−/−^ mice displayed a very low (and comparable) preference for the cold side (spending there no more than approx. 5% of the time; unpaired *t*-test; *t* = 1.077; *df* = 11; *p* > 0.05; Fig. 1f) and shank2^−/−^ mice showed an increase in distance walked on the cold side compared to WT (WT vs. Shank2^−/−^; 8.5 ± 2.1% vs. 15.3 ± 5.8%; unpaired *t*-test; *t* = 2.469; *df* = 11; *p* < 0.05; Fig. 1g). Surprisingly, this was not the case in the thermal preference test-hot (45 °C): Shank2^−/−^ mice displayed a latency to escape not significantly different from WT (unpaired *t*-test; *t* = 1.468; *df* = 11; *p* > 0.05; Fig. 1h), a comparable preference (in this case, approx 40%; unpaired *t*-test; *t* = 2.076; *df* = 11; *p* > 0.05; Fig. 1i) and a comparable distance walked on the hot side (unpaired *t*-test; *t* = 0.2011; *df* = 11; *p* > 0.05; Fig. 1j), indicating that the hot-test produced a much lower overall discomfort to either genotype than the cold test.

In the tactile allodynia test, a small piece of adhesive tape was glued to the hindpaw of each mouse and the number and latency of the attempts to remove it was measured. Interestingly, Shank2^−/−^ mice display a significant increase in the numbers of removal attempts compared to WT mice (WT vs. Shank2^−/−^; 3.5 ± 1.0 vs. 6.7 ± 1.6; unpaired *t*-test; *t* = 3.997; *df* = 10; *p* < 0.01; Fig. 1k), a significant decreased latency to notice the adhesive tape (WT vs. Shank2^−/−^; 462.5 ± 163.4 s vs. 230.8 ± 88.7 s; unpaired *t*-test; *t* = 2.997; *df* = 10; *p* < 0.05; Fig. 1l) and shank2^−/−^ mice showed an increase in distance walked compared to WT (WT vs. Shank2^−/−^; 132.8 ± 28% vs. 238.6 ± 105.7%; unpaired *t*-test; *t* = 2.367; *df* = 11; *p* < 0.05; Fig. 1m).

Finally, we investigated if Shank2^−/−^ mice may experience discomfort from the cage floor texture (in a form of tactile allodynia [44]). To this aim, the cage was divided in two parts, one covered with rough sandpaper, the other smooth but comparable in color and smell. Interestingly, Shank2^−/−^ mice show a significant increase in latency to escape compared to WT mice (WT vs Shank2^−/−^; 6.5 ± 6.6 s vs. 17.6 ± 11.6 s; unpaired *t*-test; *t* = 2.317; *df* = 13; *p* < 0.05; Fig. 1n), however not in preference for any side compared to WT mice (unpaired *t*-test; *t* = 0.4200; *df* = 11; *p* > 0.05; Fig. 1o) or distance walked on the rough side (unpaired *t*-test; *t* = 0.3082; *df* = 11; *p* > 0.05; Fig. 1p).

Taken together, these data show that Shank2^−/−^ mice display an increased response to noxious chemical stimuli (PFA injection, subacute phase) and a selective hypersensitivity to cold stimulus and a sensory-specific sensitivity to mechanical stimuli at baseline.

### A distinct Shank2^high^ neuronal subpopulation in dorsal and ventral spinal cord

**Fig. 2:**
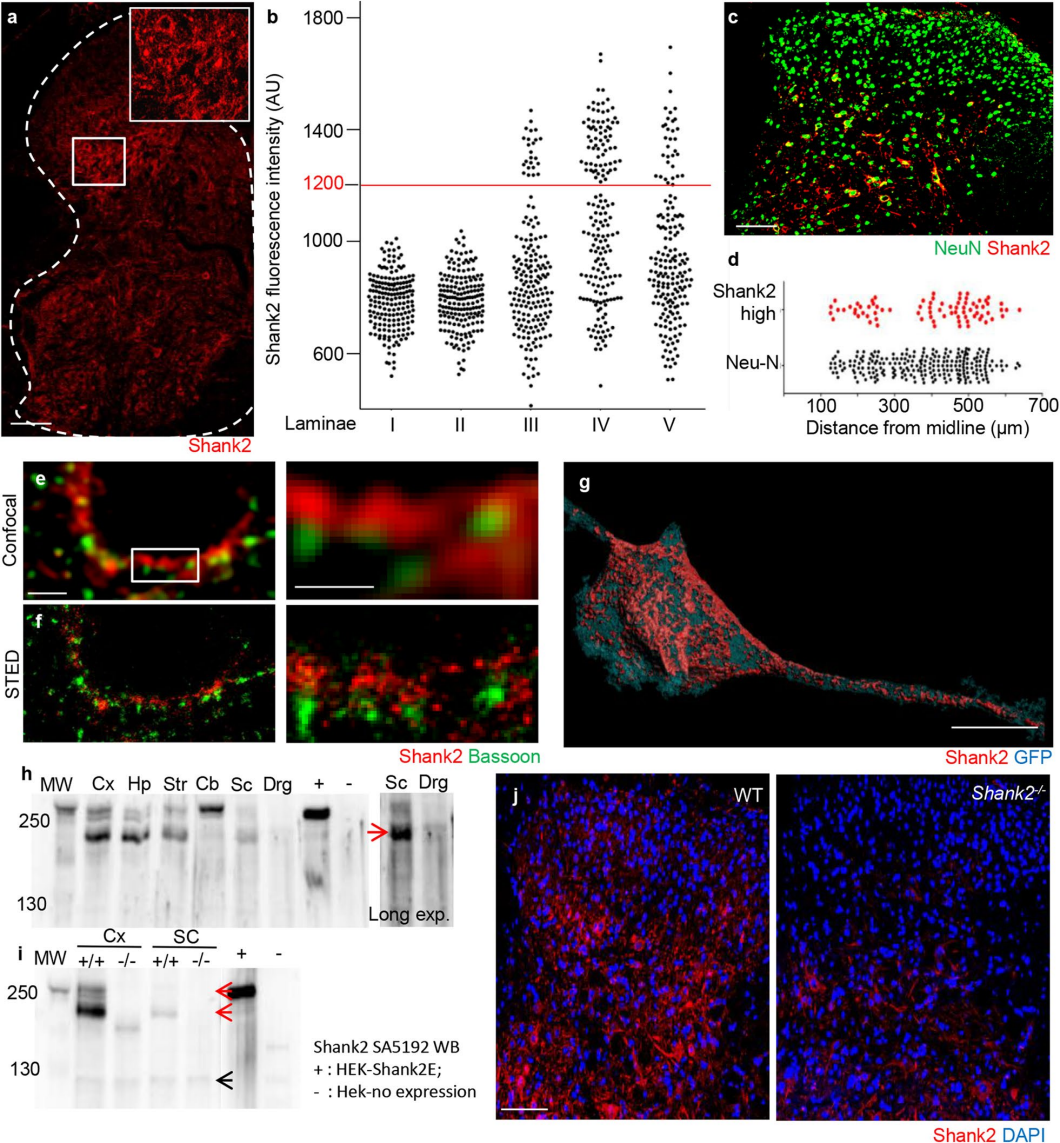
High Shank2 expression identifies a distinct neuronal subpopulation in the spinal cord. Identification of shank2 expression in the spinal cord. **a** and **b** Shank2 IF staining revealed a synaptic pattern around cells in the dorsal and ventral horn, with a subset of Shank2 high expression neurons in laminae III, IV and V (mean intensity; 785 ± 99, 788 ± 100, 892 ± 241, 1097 ± 256, 941 ± 235, Laminae I–V respectively); (N = 3; n = Laminae I–III: 180; Laminae IV–V: 181 neurons), scale bar: 100 µm. **c** and **d** These Shank2^high^ expressing neurons were further divided into location from midline in laminae III, IV and V (median; 425 ± 138 vs. 375 ± 122 Shank2^high^ vs. Neu-N; (N = 3; n = Shank2^high^: 78; Neu-N + : 155 neurons), scale bar: 50 µm. **e** and **f** High magnification confocal pictures reveal a continuous band of Shank2 around cells, however STED imaging shows the distinct punctuate patterns of Shank2 colocalised with Bassoon; (N = 3), scale bar E: 1 µm; scale bar F: 500 nm. **g** Sparse labeling of neurons in the dorsal laminae was performed by AAV9-CMV-GFP intraspinal injection, combined with Shank2 IF staining. Shank2 is highly distributed on cell-body and dendrites; (N = 3), scale bar: 1 µm. **h** Shank2 Western blot on cortex (Cx), hippocampus (Hp), striatum, (Str), cerebellum (Cb), spinal cord (Sc), dorsal root ganglia (Drg), positive control ( +) and negative control ( −), reveals 4 isoforms in the spinal cord located at 220–240, 160, 140 and 100 kD. The isoforms 160 and 100 were also detectable in other CNS regions like cortex, hippocampus, cerebellum, and Dorsal root ganglia. The cerebellum also showed the 220–240 isoform which was not found in cortex, hippocampus or DRG; (N = 3). **i**, **j** IF staining and western blot of WT and Shank2^−/−^ mice show an almost complete loss of Shank2^high^ expressing neurons in laminae III, IV and V, this loss corresponds to the loss of MW isoforms at 240 and 160 kD, the highest and lowest MW were still expressed; (N = 3), scale bar J: 100 µm

In order to mechanistically investigate the origin of the abnormal nociception in Shank2^−/−^ mice, we first analysed the expression pattern of Shank2 in the spinal cord of WT animals. Immunostaining of spinal cord sections with the anti-Shank2 SA5192 antiserum revealed a punctuated pattern distributed throughout the gray matter, coherent with Shank2 synaptic localization (Additional file 1: Fig. S1). Notably, Shank2 + puncta appeared to delineate a subset of neurons scattered in dorsal laminae III, IV and V (as well as in the ventral horn; Fig. 2a) characterized by very dense and substantially more intense Shank2 immunoreactivity. We considered the distribution Shank2 immunostaining intensity of neurons in laminae I and II, which topped at 1000 au of intensity, and we introduced a 200 au margin and we defined all neurons with averaged Shank2 immunostaining intensity larger than 1200 as Shank2^high^ neurons (see Methods for the operational identification of Shank2^high^ neuron; Fig. 2a-b and Additional file 1: Fig. S1). Since the identification of the boundaries of laminae II based on cytoarchitectonics may be subject to artifacts, we used the binding of the IB4 lectin (which recognizes a subset of nociceptive fibers and delimitates the middle/outer part of laminae II; [49–51]). We found that approx. 90% of Shank2^high^ cells (mainly large neurons) were located in laminae below the IB4 boundary (Additional file 1: Fig. S2a, blue arrows) and only a few, small neurons defined as Shank2^high^ were located within the IB4 ribbon (white arrow). Thus, these findings are in good agreement with the localization of the Shank2^high^ neurons based on cytoarchitectonics. Supporting the immunolabeling result, expression data from the adult spinal cord in the Allen Spinal Cord Atlas (http://mousespinal.brain-map.org/imageseries/show.html?id=100026736) revealed a subpopulation of Shank2 high-expressing cells intermingled with other dorsal and ventral spinal cord neurons. The dorsal population of Shank2^high^ neurons could be further distinguished into two subgroups based on the medio-lateral localization of the neurons: one (smaller) located medially and one (larger) located more laterally within the laminae III, IV and V (Fig. 2c, d).

Examined at high magnification, Shank2^high^ neurons showed Shank2 immunolabeling as an almost continuous band surrounding the cell body and proximal dendrites. Nevertheless, when samples immunostained for Shank2 (and synaptophysin) were imaged using STED [52] at 60-nm-lateral resolution (Additional file 1: Fig. S2f), the continuous Shank2 staining in Shank2^high^ neurons was resolved into a series of closely juxtaposed Shank2 clusters, of variable size, facing synaptophysin-positive presynaptic terminals (Additional file 1: Fig. S2b, c). Furthermore, STED images revealed that on Shank2^high^ neurons each Shank2 cluster matched a single Bassoon-positive release site (Fig. 2e, f and Additional file 1: Fig. S2d, e). Thus, in Shank2^high^ neurons, Shank2 is distributed in high-density clusters involved in synaptic contacts.

In order to show the expression of Shank2 in Shank2^high^ neurons in compartments distinct from the cell body, we performed a sparse-labelling of dorsal spinal neurons by injecting in the spinal cord 200 nl of AAV9-CMV-GFP suspension; 15 days after injection we selected for analysis GFP-positive Shank2^high^ neurons. Confocal stacks were acquired at high magnification over the cell body and dendrites of the selected neurons and reconstructed in 3D rendering (Fig. 2g). Shank2 clusters were distributed also on dendrites, although at reduced density compared to the cell body, confirming that Shank2^high^ neurons have Shank2-enriched synapses across the whole neuronal structure.

The Shank2 pre-mRNA transcript is known to undergo extensive alternative splicing, giving rise to multiple protein isoforms [29, 53]. We investigated the isoform expression in spinal cord homogenates by WB (together with cortical, hippocampal, striatal, cerebellar, dorsal root ganglia samples, using overexpressed full-length Shank2E-GFP as positive control and untransfected HEK-cells protein extract as negative) and probed with the SA5192 polyclonal antibody against the C-terminal domain (shared by all Shank2 isoforms; [29]) displayed one high-MW isoform (a doublet at approx. 220–240 kDa; attributable to the ankyrin-repeats-containing Shank2E isoform; also detectable in cerebellum hippocampus, cortex and striatum) and one more isoforms at 160–180 KDa (corresponding to Shank2A) and expressed in cortex, hippocampus, striatum and constituting the main expressed isoform in spinal cord (but poorly expressed in cerebellum; Fig. 2h); a very low-abundance isoform was detected at approx. 130 kDa, (possibly Shank-2B; Fig. 2h, i). We also verified the expression of Shank2 in DRG, since other Shank family members are expressed in DRG cells and are directly involved in somatosensation [14, 54]. Notably, even after prolonged exposure, only barely visible bands appeared with MW of approx. 160–180 KDa from DRG homogenates, (Fig. 2h). The immunostaining of DRG sections with the anti-Shank2 antibody produced only a low-intensity staining in a small number of DRG neurons (Additional file 1: Fig. S2g). Iimmunoblots of the homogeneates of cortex and spinal cord of WT and Shank2^−/−^ mice (along with overexpressed Shank-2E-GFP as reference; Fig. 2i) were characterized by the loss of the 240 and 180 kDa isoforms in cortex and the complete loss of the 180KDa isoform in the spinal cord (the 240 KDa isoform being not expressed in spinal cord; Fig. 2i, red arrows); only a residual, low-abundance lowest MW isoforms was still detectable (black arrow).

Immunostaining of spinal cord samples from Shank2^−/−^ mice also revealed the almost complete loss in Shank2 immunoreactivity (Fig. 2j, comparable to the largely complete loss of Shank2 immunoreactivity in cortical samples, Additional file 1: Fig. S3), with limited residual immunopositive signal in the cell body of scattered neurons in deep laminae. Together with the WB assays, the immunolabelling of cortex and spinal cord confirmed the almost complete loss of immunoreactivity for Shank2 in Shank2^−/−^ mice.

**Fig. 3:**
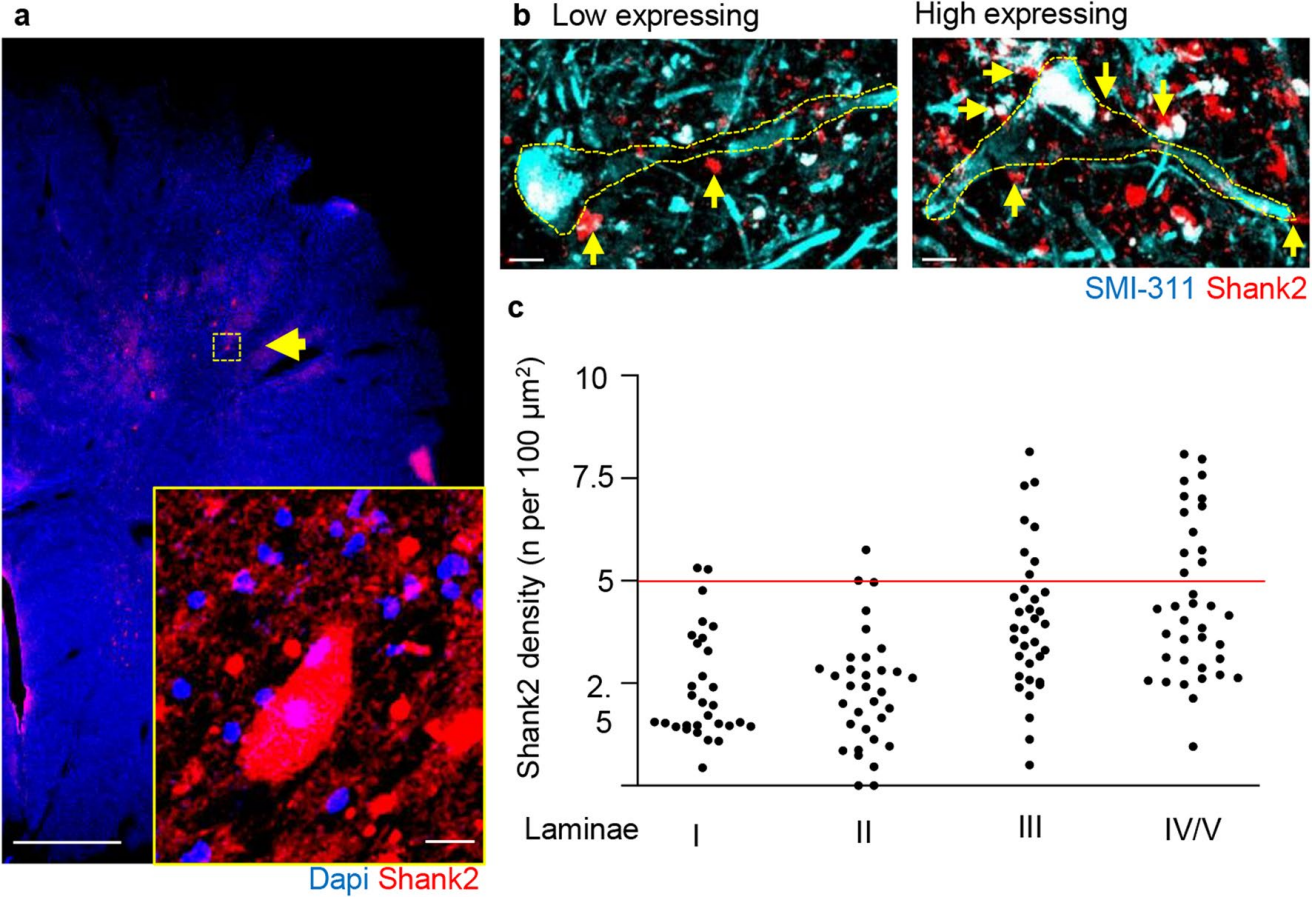
Shank2 expression in post-mortem human spinal cord tissue shows similar distribution compared to mice. Identification of shank2 expression in post-mortem human spinal cord **a** Clarity and IF staining of Human spinal cord samples reveals a subset of Shank2^high^ expressing cells in laminae III, IV and IV (white arrows). N = 5, scale bar overview: 500 µm; scale bar insert: 20 µm. **b** and **c** IF staining and high magnification imaging of Human spinal cord samples revealed expression of Shank2 across the cell body and dendrites, Laminae III and IV have neurons with a higher density of Shank2 expression on both cell body and dendrites like neurons in mouse spinal cord; (N = 3; n = Laminae I: 30; Laminae II: 33; Laminae III: 35; Laminae: 37 neurons), scale bar: 10 µm. Data shown as scatterplots

We sought to verify if Shank2^high^ were also detectable in human spinal cord: the immunostaining of postmortem human spinal cord (performed after hydrogel inclusion according to the CLARITY method modified for human samples; [55]) revealed a Shank2 expression in neurons, with a similar distribution of Shank2^high^ and Shank2^low^ across various laminae compared to mice (Fig. 3a–c). IB4 binding was erratic on these human samples, and it was not possible to use it for confirming the anatomical location of laminae II; therefore, the human anatomical data, although in good agreement with the murine data, must be interpreted with caution. Thus, Shank2 is expressed at substantial levels in the mouse and human spinal cord, and in particular in a previously unrecognized subpopulation of spinal cord neurons and it is no longer detectable in Shank2^−/−^ mice.

### Shank2^high^ neurons are a subpopulation of glycinergic interneurons in dorsal laminae

**Fig. 4:**
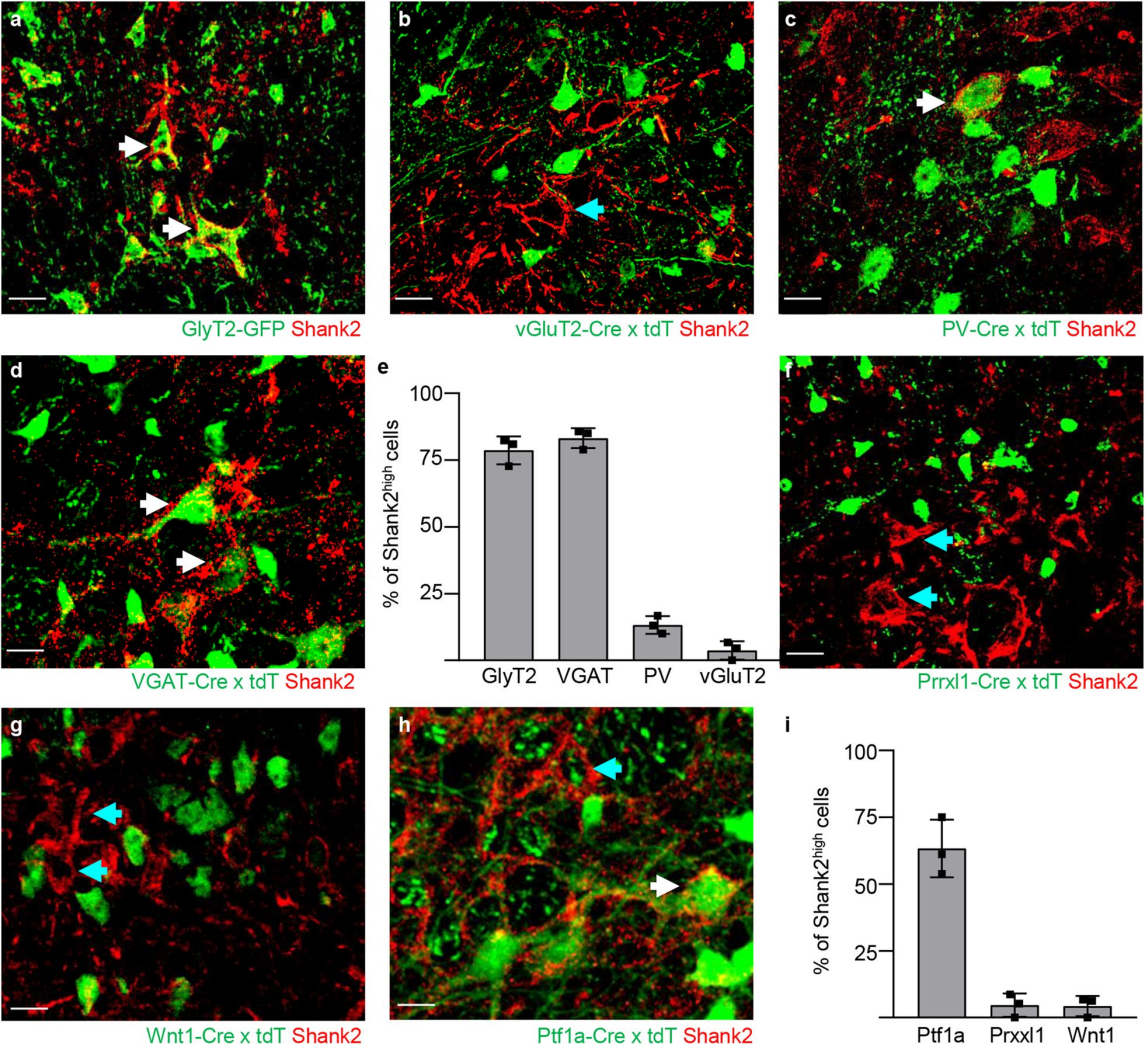
Shank2^high^ cells are mainly glycinergic interneurons. A set of mice with genetically labelled neuronal subpopulations were used to investigate in which type of cell Shank2 was mainly expressed. **a**–**e** GlyT2-GFP, vGluT2-Cre; ROSA26-Tomato, VGAT-Cre; ROSA-Tomato and PV-Cre; ROSA26-Tomato mice were used to identify inhibitory and excitatory neurons in the dorsal horn of the spinal cord. Staining spinal cord sections with Shank2 revealed the inhibitory nature of Shank2 cells (96.6 ± 0.4% vs. 2.4 ± 0.8% VGAT + vs. GlyT2 + ; white arrows in panel **d** vs. blue arrow in panel **b**), with a high number of Shank2 expressing neurons on GlyT2 + cells (82.4 ± 4.2%; white arrows in panel **a** and a lower amount of Shank2 expressing neurons on PV + cells (12.9 ± 0.4%; white arrows in panel **c**). High expression of Shank2 was mainly observed in GlyT2 + cells compared to PV + cells (70% vs. 20%); (N = 3) scale bar: 20 µm. **f**–**i** Prrxl-Cre;Tomato, Wnt1-Cre;Tomato and ptf1a-Cre;Tomato mice were used to identify a subset of transcription factors regulating excitatory and inhibitory phenotypes. Staining spinal cord sections of these mice resulted in a low fraction of Shank2 expressing neurons on prrxl1 + and Wnt1 + cells (blue arrows in panel **f** and **g**) and a high number of Shank2 expressing neurons on ptf1a + cells (66 ± 12.3%; white arrows in panel **h**); (N = 3) scale bar: 20 µm. Data shown as average ± SD

In order to establish the neurochemical identity of the Shank2^high^ neurons, we immunostained for Shank2 an array of spinal cord samples from mice in which distinct spinal cord neuronal subpopulations have been genetically-labelled: vGlut2-Cre;ROSA26-Tomato to mark excitatory neurons, GlyT2-GFP, VGAT-CRE;ROSA26-Tomato or PV-CRE;ROSA26-Tomato to label all or part of inhibitory interneurons. In laminae III and IV, only 3.7 ± 3.4% of Shank2^high^ colocalized with Tomato + neurons in vGlut2-Cre;ROSA26-Tomato mice, suggesting the inhibitory nature of Shank2^high^ cells (Fig. 4b, e). In fact, 78.7 ± 5.2% of Shank2 neurons were GFP + in GlyT2-GFP mice (Fig. 4a, e) and virtually all (83.2 ± 3.7%) Shank2^high^ neurons was Tomato + in VGAT-CRE;ROSA26-Tomato (Fig. 4d, e). In PV-CRE/tdTomato mice, a small fraction (13.2 ± 3.3%) of Shank2^high^ neurons corresponded to PV-positive interneurons (Fig. 4c, e), almost all in the medial population (located in the *nucleus proprius* of the spinal cord). Nevertheless, Shank2^high^ cells represented only 71.1 ± 4.1% of glycinergic interneurons (up to 30% of GlyT2-GFP cells and more than 80% of PV + neurons were Shank2^low^, data not shown). We further characterized the transcriptional and developmental identity of Shank2^high^ neurons. In agreement with their inhibitory nature, Shank2^high^ neurons did not correspond to Prrxl1 + cells (4.6 ± 4.4%; excitatory interneurons involved in the processing of nociceptive inputs [56]; Fig. 4f, i) in Prrxl-Cre/TdTomato mice or to neurons originated from dorsal, Wnt1-positive progenitor populations (4.3 ± 3.7%; in Wnt1-cre; ROSA26-Tomato double tg mice [57]; Fig. 3g, i). On the other hand, a significant fraction (63.3 ± 10.7%; Fig. 4h, i) of Shank2^high^ neurons correspond to Tomato + cells in Ptf1a-Cre;ROSA26-Tomato mice; in fact, Ptf1a transcriptionally regulates the development of inhibitory interneurons phenotype and neurochemistry [58].

Taken together, these data prove that Shank2^high^ neurons represent a previously unrecognized subpopulation of inhibitory glycinergic/GABAergic interneurons in the sensory areas of the spinal cord.

### Shank2+ synapses in Shank2^high^ neurons receive multiple mechanosensory and propriospinal input

We then set out to investigate if the Shank2 clusters on Shank2^high^ neurons constituted the specialized postsynaptic counterpart of a specific input, e.g. from dorsal root ganglia afferents [35, 59].

**Fig. 5:**
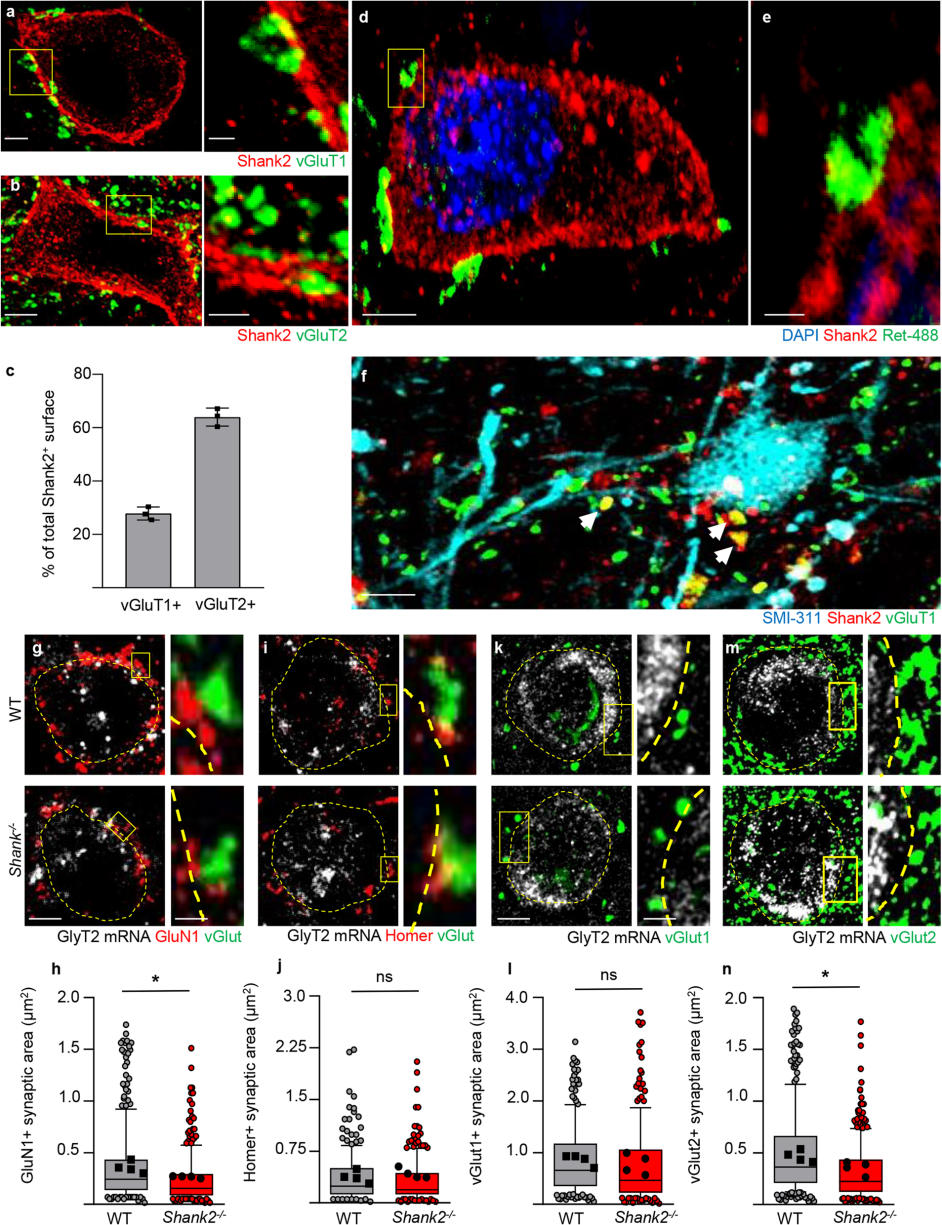
Propriospinal and mechanosensory input on Shank2^high^ expressing neurons. Synaptic input on Shank2 high expressing glycinergic neurons. **a**–**c** Excitatory pre-synaptic input on Shank2 post-synaptic structures show both a vGlut1 (27.8 ± 7.1% of total Shank2 area) and vGlut2 (64.0 ± 9.5% of total Shank2 area) input on glycinergic interneurons; (N = 3) scale bar overview: 2.5 µm; insert: 1 µm. **d** and **e** Detection of GFP positive buttons on Shank2 positive terminals in an c-RET-GFP mouse to show mechanosensory afferents on Shank2^high^ expressing neurons; (N = 3) scale bar D: 5 µm; scale bar: E. **f** IF staining on postmortem human spinal cord reveals that Shank2 post-synaps receive vGluT1 pre-synaptic input (36.9 ± 14.1% of Shank2 positive puncta; white arrows); (N = 3) scale bar: 10 µm. **g**–**j** post-synaptic structure GluN1 shows a significant decrease in size on glycinergic neurons in Shank2^−/−^ mice (*p* < 0.0001), however this decrease was not observed in the post-synaptic structure Homer (*p* > 0.05); (N = 4; panel **h**; n = WT: 299; shank2^−/−^: 265 synapses; panel **j**; n = WT: 231; Shank^−/−^: 223 synapses) scale bar overview: 5 µm; insert: 2 µm. **k**–**n** Pre-synaptic structure vGlut1 was not affected on glycinergic cells in Shank2^−/−^ mice (*p* > 0.05), interestingly vGlut2 shows a significant decrease in size on glycinergic neurons in Shank2^−/−^ mice (*p* < 0.0001); (N = 4; panel **l**; n = WT: 306; shank2^−/−^: 297 synapses; panel **j**; n = WT: 211; Shank.^−/−^: 208 synapses) scale bar: 5 µm; insert: 2 µm. Data shown as average ± SD and box and whiskers (10–90 percentile). *p < 0.05

First, we co-immunostained spinal cord sections for Shank2 and for the presynaptic proteins vGluT1 (a marker of myelinated low-threshold mechanosensitive sensory fibers including proprioceptive afferents) and vGluT2 (representing local spinal glutamatergic interneurons and at lower levels high threshold (nociceptive) afferent fibers; [60]) and we assessed if Shank2 + structures were preferentially juxtaposed to one of the two terminals. Interestingly, Shank2 immunopositivity in Shank2^high^ was not restricted to one of the two types of input: vGlut1 + presynaptic terminals covered 27.9 ± 2.4% of Shank2 + cluster area, whereas vGlut2 + terminals covered no less than 64.0 ± 3.4% (Fig. 5a–c).

We sought to confirm the nature of the projections to Shank2 + synapses more precisely. First, we injected fluorescently labelled Cholera Toxin B subunit (CTB-488) into the hairy skin to label myelinated cutaneous afferents (thus excluding non-myelinated nociceptive fibers); central terminals labelled by this approach were distributed in a narrow column elongated along the dorso-ventral axis of the spinal cord grey matter [35]. Notably, even when a comparatively small area of the hindlimb skin was injected, several CTB-positive presynaptic boutons were detected on the cell body and proximal dendrites of Shank2^high^ glycinergic interneurons, in close apposition to Shank2 clusters (Additional file 1: Fig. S4a–c).

Second, we used c-RET-GFP transgenic mouse [28] to track the central processes of mechanosensory afferents [61]. Also in this case, numerous GFP + terminals were identified impinging on Shank2^high^ neurons; GFP + presynaptic terminals were found to be juxtaposed to large Shank2 postsynaptic clusters (Fig. 5d, e). Finally, immunostaining on postmortem human spinal cord revealed that the post-synaptic Shank2 terminals receive pre-synaptic input of vGluT1, 36.9 ± 14.1% of Shank2 synapses were positive for vGlut1 (Fig. 5f), which was comparable to the mouse data.

Thus, Shank2^high^ receive direct somatosensory input but Shank2 + synapses do not correspond to a distinct type of input.

### Neuronal architecture of the spinal cord is unaffected by Shank2^−/−^

Having established the existence and the nature of a new Shank2^high^ subpopulation of glycinergic interneurons, we set out to investigate how the loss of Shank2 in Shank2^−/−^ mice may affect these interneurons and generate the pain-hypersensitivity phenotype observed in behavioural tests. Since several transgenic mouse lines with sensory or nociceptive phenotypes are characterized by disruption or selective loss of neuronal subpopulations [41, 62] we verified that loss of Shank2 did not result in any gross disturbance of spinal cord architecture. In fact, Two-way ANOVA (Bonferroni corrected) revealed that the overall density of neurons (NeuN+; F_(1.16)_ = 3.977; *p* > 0.05; Additional file 1: Fig. S5a, b) and in particular of inhibitory neurons (Pax2+; F_(1.16)_ = 0.3959; *p* > 0.05) in the dorsal laminae of the spinal cord was comparable in WT and Shank2^−/−^ (Additional file 1: Fig. 5c, d). Furthermore, unpaired t-test revealed that the density of excitatory interneurons of laminae II (Protein kinase C gamma+ (PKC-γ+); t = 2.296; *df* = 4; *p* > 0.05) was comparable in WT and Shank2^−/−^ (Additional file 1: Fig. 5e, f). Likewise, the number of neurons expressing GlyT2 mRNA was similar in WT and Shank2^−/−^ mice (F_(1.16)_ = 0.2588; *p* > 0.05; Additional file 1: Fig. 5g, h).

Finally, we determined the density of inhibitory synapses across the dorsal laminae; no significant difference has been found between WT and Shank2^−/−^ in the number of synapses per area (synaptic density) for GlyT2+ puncta (Two-way ANOVA: F_(1.30)_ = 0.8204; *p* > 0.05; Additional file 1: Fig. S6a, b), VGAT+ puncta (F_(1.20)_ = 0.004215; *p* > 0.05; Additional file 1: Fig. S6c, d) and gephyrin+ puncta (F_(1.48)_ = 3.626; *p* > 0.05; Additional file 1: Fig. S6e, f). Thus, deletion of Shank2 did not cause any obvious changes in the overall architecture of the dorsal horn.

### Loss of Shank2 disrupts NMDAR clustering in excitatory synapses on glycinergic interneurons

Since Shank2 is a core scaffold protein in glutamatergic synapses, we reasoned that excitatory synapses on Shank2^high^ glycinergic interneurons may suffer substantial postsynaptic (and/or presynaptic) alterations in Shank2^−/−^ mice. We identified glycinergic interneurons by the detection of GlyT2 in situ hybridization and co-immunostained the sections for postsynaptic proteins and receptors. This approach provides a representative value of the synaptic inputs, within the limitation imposed by the consideration of the cell body only (dendrites are not easily visible with this approach). Because of the protease treatment required for mRNA detection, only a restricted number of antibody-antigen couples (whose antigens were protease-resistant) could be employed. In particular, we considered the GluN1 NMDAR subunit and the PSD scaffold protein Homer. Interestingly, the number of GluN1 and Homer post-synaptic structures on GlyT2 interneurons was comparable in WT vs Shank2^−/−^ mice (95.3 ± 17.5 vs. 107.4 ± 31 per 100 µm WT vs. Shank2^−/−^; unpaired *t*-test; *t* = 1.034; *df* = 6; *p* > 0.05 for GluN1; 80.1 ± 20.2 vs. 104.7 ± 25.8 per 100 µm WT vs. Shank2^−/−^; unpaired *t*-test; *t* = 1.010; *df* = 6; *p* > 0.05; for Homer) indicating that the number of synapses and their qualitative composition were not affected by Shank2 loss. However, GluN1 clusters were significantly smaller in glycinergic interneurons of Shank2^−/−^ mice compared to WT mice (WT vs. Shank2^−/−^; 0.36 ± 0.06 µm^2^ vs. 0.27 ± 0.01 µm^2^; unpaired *t*-test; *t* = 3.229; *df* = 6; *p* < 0.05; Fig. 5g, h). Remarkably, this reduction in size was not observed for Homer (WT vs. Shank2^−/−^; 0.36 ± 0.36 µm^2^ vs. 0.32 ± 0.32 µm^2^; unpaired *t*-test; *t* = 0.5888; *df* = 4; *p* > 0.05; Fig. 5i, j).

Since Shank2 proteins may regulate trans-synaptic signaling, we also took into consideration the number and the size of presynaptic excitatory boutons. To this aim, glycinergic interneurons were identified by GlyT2 in situ hybridization and vGlut1+ and vGlut2 + terminals by immunostaining. The number of vGlut1 and vGlut2 terminals on GlyT2 interneurons was comparable in WT and Shank2^−/−^ mice (16.3 ± 5.2 vs. 17.8 ± 7.8 per 100 µm WT vs. Shank2^−/−^; unpaired *t*-test; *t* = 0.3373; *df* = 6; *p* > 0.05 for vGlut1; 59.2 ± 17.7 vs. 64.2 ± 18.8 per 100 µm WT vs. Shank2^−/−^; unpaired *t*-test; *df* = 6; *p* > 0.05 for vGlut2); however, vGluT2 terminals were significantly smaller in Shank2^−/−^ mice compared to WT mice (WT vs. Shank2^−/−^; 0.47 ± 0.06 µm^2^ vs. 0.36 ± 0.06 µm^2^; unpaired *t*-test; *t* = 2.599; *df* = 6; *p* < 0.05; Fig. 5m, n). This difference was not observed in the vGluT1 terminals (WT vs. Shank2^−/−^; 0.86 ± 0.69 µm^2^ vs. 0.79 ± 0.78 µm^2^; unpaired *t*-test; *t* = 0.9864; *df* = 6; *p* > 0.05; Fig. 5k, l).

Taken together these data show that loss of Shank2 does not affect the number of excitatory synapses on glycinergic interneurons but causes the selective decrease of synaptic NMDAR content and in the size of propriospinal glutamatergic presynaptic terminals. Thus, Shank2 loss appears to selectively weaken glutamatergic input and plasticity on glycinergic interneurons.

### Reduced activation of glycinergic interneurons in Shank2^−/−^ mice is associated with increased excitation of dorsal laminae interneurons upon nociceptive stimulation

**Fig. 6:**
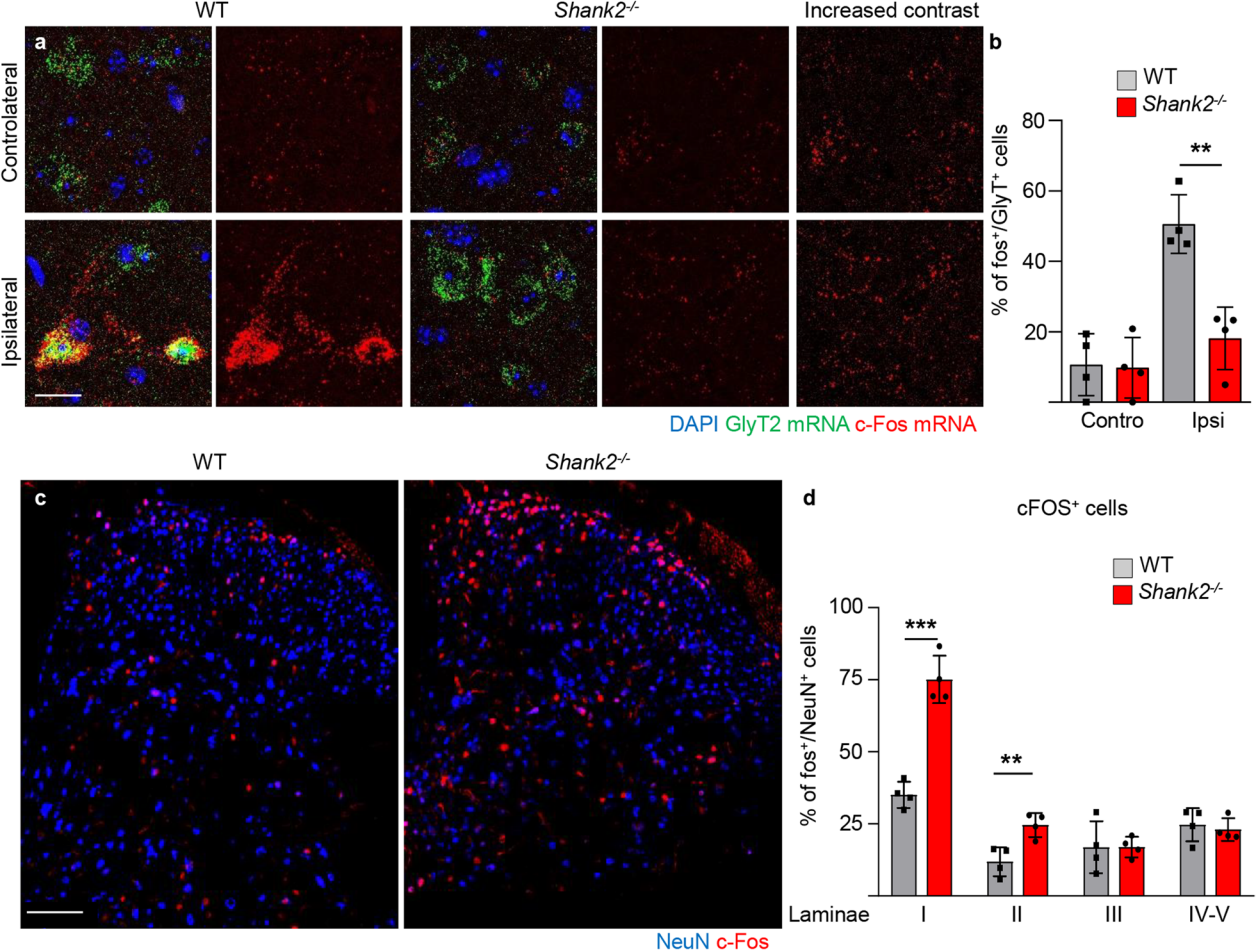
Reduced activity in glycinergic interneurons is associated with increased activity of interneurons in the dorsal laminae I and II. Activation patterns in glycinergic interneurons and interneurons in dorsal laminae upon activation. Mice were treated with low concentration of Formalin and c-fos levels were measured 120 min later (**a**, **b**) Saline injection resulted in no difference in c-fos levels in glycinergic cells between WT and Shank2^−/−^ mice (*p* > 0.05). Upon Formalin injection c-fos was significantly higher in WT vs Shank2^−/−^ mice in glycinergic cells (*p* < 0.01); (N = 4) scale bar: 20 µm. **c** and **d** The reduced activity of glycinergic cells in Shank2^−/−^ mice in turn significantly increased the overall c-fos expression in the Shank2.^−/−^ in laminae I (*p* < 0.001), Laminae II (*p* < 0.01) but no difference in laminae III and IV–V (*p* > 0.05; *p* > 0.05) upon formalin injection; (N = 4) scale bar: 100 µm. Data shown as average ± SD. **p < 0.01; ***p < 0.001

Next, we investigated if the disturbance in the structure of synaptic inputs to Shank2^high^ interneurons affected their activation during nociceptive stimulation. To this aim, WT and Shank2^−/−^ mice were injected with Formalin (or with saline) in the plantar aspect of the right hindpaw (as in the Formalin test) and sacrificed 120 min later (90 min after the onset of the phase-II). We used single-mRNA molecule in situ detection to simultaneously identify glycinergic interneurons (using GlyT2 mRNA) and c-fos mRNA expression as a marker of neuronal activity (the use of c-fos immunostaining was precluded by the protease treatment necessary for the identification of GlyT2 mRNA). Two-way ANOVA revealed a significant effect in the treatment groups (F_(3,9)_ = 21.67; *p* = 0.0002). Post-hoc analysis (Bonferroni corrected) revealed that indeed, the number of c-fos + /GlyT2 + neurons was very low at baseline and comparable in WT and Shank2^−/−^ samples (WT vs. Shank2^−/−^; 10.7 ± 4.7% vs. 9.8 ± 5.2%; Two-Way ANOVA; *df* = 9; *p* > 0.05, Fig. 6a, b); however, upon Formalin challenge, the number of double-positive interneurons increased strongly in WT animals but much less so in Shank2^−/−^ (WT vs Shank2^−/−^; 50.6 ± 8.3% vs. 18.1 ± 8.9%; Two-Way ANOVA; *df* = 9; *p* < 0.01; Fig. 6a, b) implying a failure in GlyT2+ neuron activation upon nociceptive stimulation in the Shank2^−/−^ mice.

We then sought to verify if the observed decrease in inhibitory interneurons activity would result in the overall increase in excitation in the dorsal-horn circuitry of Shank2^−/−^ mice. We considered spinal cord sections obtained 120 min after a Formalin challenge in the right hindpaw; in order to monitor the large-scale activation of neurons in the dorsal spinal cord, the samples immunostained for NeuN (to identify all neurons) and c-Fos and the ratio of c-Fos+/NeuN+ cells was annotated according to the spinal cord lamina. Two-way ANOVA revealed a significant effect in treatment groups (F_(1,48)_ = 62.82; *p* < 0.0001). Post-hoc analysis (Bonferroni corrected) revealed that, in saline-injected WT mice, c-Fos+ neurons were detectable in very low numbers across dorsal spinal cord (2.2 ± 1.1% in laminae I) and in saline-injected Shank2^−/−^ mice the percentage of c-fos+ neurons were comparable to WT mice (4.5 ± 2.7% of NeuN+ in laminae I; *p* > 0.05). Formalin injection caused a much larger elevation of c-Fos+ neurons in Shank2^−/−^ vs WT in laminae I (WT vs. Shank2^−/−^; 35.1 ± 4.5% vs. 75.1 ± 8.2%; Two-Way ANOVA; *df* = 24; *p* < 0.0001; Fig. 6c, d) as well as in laminae II (WT vs. Shank2^−/−^; 11.9 ± 5.0% vs. 24.6 ± 4.2%; Two-Way ANOVA; *df* = 24; *p* < 0.001; Fig. 6c, d); but no difference was found in laminae III, IV and V (WT vs. Shank2^−/−^; Laminae III: 16.9 ± 9.0% vs. 16.9 ± 3.6%; WT vs. Shank2^−/−^; *p* > 0.05; Laminae IV-V: 24.7 ± 5.8% vs. 23.0 ± 3.9%; Two-Way ANOVA; *df* = 24; *p* > 0.05; Fig. 6c, d). A comparable result was obtained when the number of cells positive for c-Fos mRNA (by in situ hybridization) was measured in the same groups (Two-way ANOVA: F_(1.24)_ = 35.60; *p* < 0.0001); post-hoc analysis (Bonferroni) showed a significant difference between Shank2^−/−^ versus WT in laminae I (WT vs. Shank2^−/−^; 4.0 ± 0.9 vs. 8.7 ± 1.4; per 10^4^ µm^2^; *p* < 0.0001; Additional file 1: Fig. S7a, b), underscoring the robustness of the dataset.

Taken together, these data suggest that the disruption of the excitation of inhibitory interneurons in Shank2^−/−^ mice results in a decreased excitation of glycinergic inhibitory interneurons upon acute chemical pain and, in turn, to the increased activation of neurons in the dorsal horn following a nociceptive stimulus.

## Discussion

Our data show that high levels of Shank2 expression identifies a subpopulation of glycinergic inhibitory interneurons located in the dorsal horn of the spinal cord, receiving inputs from somatosensory afferents; Shank2 loss leads to a decrease in excitatory synapses onto glycinergic interneurons and in net increase of excitation in the dorsal horn, correlated with increased sensitivity to chemically induced pain.

The proposed mechanism involved in pain hypersensitivity of Shank2^−/−^ mice is therefore related to dis-inhibition of spinal cord nociceptive circuits [63, 64]. A similar increase in nociception has been observed in several experimental models in which the inhibitory tone has been decreased: pharmacological blockade of glycinergic inhibition produces mechanical allodynia through the dis-inhibition of PKC-γ+ neurons [65] and silencing of glycinergic interneurons is sufficient to induce allodynia [59]. This concept has been further supported by recent data obtained in patients with loss-of-function mutations of glycine receptors or glycine transporters (leading to the clinical syndrome of hyperekplexia), who display decreased pain thresholds and amplified pain withdrawal reflexes [66]. Increased nociception as a consequence of loss of spinal inhibition has been also observed in case of loss of GABAergic inputs [67]: inactivation of PV+ inhibitory interneurons cause the appearance of tactile allodynia [68, 69], whereas GABAergic agonists produce anesthesia [70]. Within the conceptual framework of the “gate-control” theory, inhibitory inputs are well understood to prevent the runaway activation of PKC-γ+ interneurons by touch-evoked inputs as well as other excitatory interneurons in laminae II [15], which, in turn, would drive the excitation of laminae I (NK1+) interneurons. In agreement with this model, reduced excitation of glycinergic interneurons in Shank2^−/−^ mice leads to increased activity of laminae I neurons.

Abnormalities in pain processing have been previously reported in Shank2^−/−^ mice [21, 71]. Ko et al. reported of an overall decreased basal tactile perception and acute pain response in Shank2^−/−^ and induction of neuropathic or inflammatory chronic pain. Some of the reported findings agree with our own: the reduced mechanical allodynia at baseline reported by Ko is also visible in our dataset (Fig. 1d-pre) with a response comparable in the two genotypes up to day 7 in the CFA-von Frey test followed by reduced chronic pain at a later timepoint (Fig. 1d). Furthermore, Shank2^−/−^ did display a longer latency to escape in the texture preference test (Fig. 1n), implying a decrease in tactile sensitivity. In other cases, conditions are not fully comparable: the hot-plate test was performed by Ko at 55 °C, whereas 45 °C was used in this study, and we did not specifically investigate neuropathic pain, which may hinge on different long-term sensitization mechanisms [72]. Taken together, ours and previously published data suggest that nociceptive abnormalities in Shank2^−/−^ may be specific of sensory modalities and depending on multiple alterations in synaptic plasticity and circuit function.

Genetic approaches have increasingly revealed the high degree of heterogeneity in neuronal subpopulations in the dorsal spinal cord [41, 61, 62]. Recently, the genetic diversity of these populations has been demonstrated to be extensive and a large number of subpopulations have been identified in a single-cell transcriptome study [73]. Nevertheless, neuronal physiology is highly influenced by the quantity and quality of their synaptic inputs, which is strongly dependent upon the composition and architectural organization of postsynaptic structures [74, 75]. Therefore, a distinct layer of diversity may be identified once the synaptic composition of neuronal subtypes is taken into consideration. Here we provide a first proof of this concept; in fact, Shank2 distinguishes a subclass of glycinergic and parvalbumin interneurons as well as a small population of excitatory interneurons.

Since different members of the Shank family, despite their similarity, are not considered mutually redundant [19, 76], the function of Shank2^high^ cells is predicted to be heavily impacted by Shank2 loss, despite their anatomical integrity (i.e., their normal number and positioning). Of note, the Shank2 gene gives rise to several splice variants and isoforms generated using alternative promoters (ranging from 130 to 230 KDa; [29, 53, 77]). The Shank2^−/−^ model used in the present study is more precisely a deletion mutant lacking the exon 7 and this mutation should lead to the early termination and non-sense decay of the Shank2 mRNA [19]. In agreement with the cortex finding, in the spinal cord homogenate of Shank2^−/−^ mice we observe the loss of the most abundant isoforms (160–220 kDa) whereas only a low abundance, low MW isoform may still be expressed. However, the immunostaining of cortical samples from Shank2^−/−^ mice reveals a largely complete loss of immunoreactivity and the immunostaining of spinal cord from Shank2^−/−^ mice reveals an almost complete loss of immunoreactivity (any residual immunoreactivity seen in spinal cord may correspond to the low-abundance 130 KDa isoform, whose functional relevance, if any, remains to be investigated).

DRG are known to express some isoforms of Shank3, which contribute to arrange the arrays of receptors and ion channels in the peripheral projections of the ganglion cells [14, 54]. However, DRGs appear to express very little of the 160–180 KDa better known Shank2 isoform, and almost none of the 160 kDa isoform which is characteristically eliminated by the deletion of the exon 7. The immunostaining of DRG for Shank2 revealed only a small number of cells with a very faint immunopositivity, in contrast with the abundant and widespread expression of Shank3 [14]. While it is not possible to fully exclude any DRG or peripheral effect of Shank2 loss on sensory phenotypes observed in our mouse model, the limited expression of Shank2 in DRG suggest that at least a component, possibly a substantial one, of the observed phenotype is due to the loss of Shank2 within the central nervous system (CNS).

In agreement with observations in other neuronal subtypes [22, 76, 78], we find that loss of Shank2 causes the decrease in NMDAR expression in excitatory synapses on GlyT2+ interneurons. Although baseline neurotransmission in the pain processing circuit appears to be not affected (as the acute phase of the formalin test is comparable in Shank2^−/−^ mice and WT littermates), the NMDAR-dependent synaptic plasticity that is thought to underlie the second phase of the behavioural response to the formalin test [79–81] may be unbalanced, with insufficient potentiation of the inhibitory circuit. In fact, glycinergic interneurons are strongly activated in WT (as shown by the c-Fos induction), but not in the Shank2^−/−^ animals. Thus, the resulting decrease in glycinergic transmission in the pain processing circuit would cause excessive excitatory drive, as demonstrated by the increase in the number of c-fos+ neurons in laminae I and II. In fact, dysfunction or silencing of inhibitory interneurons is known to cause pain hypersensitivity in human patients and experimental animals [59, 66, 82] in particular by dis-inhibiting PKC-γ+ excitatory interneurons [65]. However, disturbances in excitatory synapses due to Shank2 loss may affect other neuronal elements of the circuit: in fact, pain induced by intrathecal administration of NDMA is reduced in Shank2^−/−^ mice, suggesting that Shank2 loss may affect differentially multiple sensory and nociceptive modalities.

Thus, circuits involved in nociception may be disturbed across multiple nociceptive stimuli and alteration of somatosensation may co-exist (as previously shown, [54, 83]). Interestingly, Shank2^−/−^ mice have been also reported to display a reduced sensitivity to the nociceptive response evoked by intrathecal injection of NMDA [21]. Since this procedure does not selectively activate one subpopulation of neurons, it is not straightforward to explain it in circuit terms. In fact, Shank2 is highly enriched in glycinergic interneurons, but it is not restricted to these cells (see Fig. 1a, b and Additional file 1: Fig. S1) and even among the Shank2^high^ cells, a fraction of excitatory neurons (VGluT2+, Prrxl1+) can be identified and their role remains unexplored. Furthermore, the dysfunction of PV interneurons in spinal cord has been directly related to mechanical but not thermal allodynia[69]; interestingly, only a fraction of PV interneurons appear to be Shank2^high^ and indeed we detect thermal but not mechanical allodynia.

Thus, the impact of Shank2 loss may affect modality-specific pain processing circuits in a distinct way, depending on the role of different cellular subpopulations.

The insufficient activation of glycinergic interneurons because of disrupted excitatory synapses observed in Shank2^−/−^ mice is a new mechanism for abnormal pain processing in autism. In fact, reported pain hyposensitivity in Shank3 mice has been linked to the loss of Shank3 in neurons in the dorsal root ganglia and the direct effect of Shank3 absence on TRPV channel expression [14]. Likewise, autism-related behavioural dysfunctions have been linked to the disturbed sensory input generated by abnormal sensory neurons in dorsal root ganglia [54, 83]. Conversely, loss of function of the ASD-associated gene Caspr2 is associated with neuropathic pain [84] through mechanisms involving increased sensitization of neurons in dorsal root ganglia. Although these reports all point to a sensory dysfunction originating in the periphery, our findings suggest that disruption of spinal cord circuits may be a strong contributor to the observed hyper- and hyposensitivity to nociceptive stimuli. Furthermore, one can speculate that the same excitation/inhibition balance disruption that is thought to underlie the ASD spectrum disorder may also manifest itself in spinal circuits to contribute to drive sensory abnormalities.

## Limitations

The findings of this study must be considered within the boundaries of a few limitations. First, we explored the nociceptive phenotype only in male mice; since ASD is more prevalent in males (3:1, [85]), our findings are relevant to the majority, but not necessarily to all, of ASD patients. Nevertheless, gender dimorphism in pain sensitivity has been reported [86, 87] and extrapolation of our results to females has to be cautious. Second, ASD patients display a large genetic heterogeneity [13] and a very diverse phenotype in nociception, ranging from hyposensitivity to hypersensitivity. Thus, our findings may not necessarily apply to ASD related to mutations in genes other than Shank2.

## Conclusion

In conclusion, we demonstrate that the Shank2 expression level characterizes a subset of inhibitory interneurons whose activation is disrupted by Shank2 loss leading to excessive nociceptive circuit activation and allodynia. Although Shank3 expression is known to be compensatory increased upon Shank2^−/−^ [19], no functional compensation takes places in glycinergic interneurons, whose excitatory synapses appear to be functionally and structurally disrupted. It remains unclear which role of Shank2 is absolutely required in these cells that cannot be compensated by other Shank family members. Our findings also suggest that treatment of nociceptive disturbances in ASD may require to be tailored to the underlying genetic cause, even when phenotypes converge, since distinctive pathophysiology may be at play in each case.

## Supplementary Information

**Additional file 1: Figure S1**: Shank2 spinal cord localisation. **Figure S2** Shank2 architecture and localisation. **Figure S3** Loss of Shank2 immunoreactivity in cerebral cortex from Shank2^−/−^ mice. **Figure S4** CTB-488 injections reveal cutaneous afferents on Shank2^high^ expressing neurons. **Figure S5** No difference in neuronal architecture in the spinal cord of Shank2^−/−^ mice. **Figure S6** No difference in inhibitory synapses in the spinal cord of Shank2^−/−^ mice. **Figure S7** Increased number of cFos mRNA + interneurons in Laminae I upon Formalin injection. **Table S1** Primary and secondary antibodies used for immunostainingFluorescent in Situ Hybridisationor for western blot. **Table S2** Full details regarding human samples. **Source data file 1** uncropped Western Blot of Shank2 in mouse tissue.

## Data Availability

All data generated or analysed during this study are included in this published article.

## Abbreviations

AAV: Adeno associated virus
ACSF: Artificial cerebrospinal fluid
ASD: Autism spectrum disorder
BSA: Bovine serum albumin
CFA: Complete Freund adjuvant
CNS: Central nervous system
CTB: Cholera toxin subunit B
DRG: Dorsal root ganglion
ECL: Enhanced chemiluminescent
GlyT2: Glycinergic transporter 2
HRP: Horseradish peroxidase
NDS: Normal donkey serum
OCT: Optimal cutting temperature
PBS: Phosphate buffered saline
PFA: Paraformaldehyde
PKC-y: Protein kinase C gamma
Prrxl1: Paired related homeobox protein-like 1
PSD: Postsynaptic density
Ptf1a: Pancreas associated transcription factor 1a
PV: Parvalbumin
RIPA: Radioimmunoprecipitation assay buffer
RT: Room temperature
STED: Stimulated emission depletion
TBS: Tris buffered saline
TDE: 2,2-Thiodiethanol
VGAT: Vesicular GABA transporter
vGluT2: Vesicular glutamate transporter 2
WT: Wild type

## References

[1] Tomchek SD, Dunn W (2007). Sensory processing in children with and without autism: a comparative study using the short sensory profile. Am J Occup Ther.

[2] Moore DJ (2015). Acute pain experience in individuals with autism spectrum disorders: a review. Autism.

[3] Duerden EG, Taylor MJ, Lee M, McGrath PA, Davis KD, Roberts SW (2015). Decreased sensitivity to thermal stimuli in adolescents with autism spectrum disorder: relation to symptomatology and cognitive ability. J Pain.

[4] Klintwall L, Holm A, Eriksson M, Carlsson LH, Olsson MB, Hedvall A (2011). Sensory abnormalities in autism. A brief report. Res Dev Disabil.

[5] Tordjman S, Anderson GM, Botbol M, Brailly-Tabard S, Perez-Diaz F, Graignic R (2009). Pain reactivity and plasma β-endorphin in children and adolescents with autistic disorder. PLoS ONE.

[6] Fründt O, Grashorn W, Schöttle D, Peiker I, David N, Engel AK (2017). Quantitative sensory testing in adults with autism spectrum disorders. J Autism Dev Disord.

[7] Riquelme I, Hatem SM, Montoya P (2016). Abnormal pressure pain, touch sensitivity, proprioception, and manual dexterity in children with autism spectrum disorders. Neural Plast.

[8] Chen C, Hung AY, Fan YT, Tan S, Hong H, Cheng Y (2017). Linkage between pain sensitivity and empathic response in adolescents with autism spectrum conditions and conduct disorder symptoms. Autism Res.

[9] Cascio C, McGlone F, Folger S, Tannan V, Baranek G, Pelphrey KA (2008). Tactile perception in adults with autism: A multidimensional psychophysical study. J Autism Dev Disord.

[10] Clarke C (2015). Autism spectrum disorder and amplified pain. Case Rep Psychiatry.

[11] Loades ME (2015). Evidence-based practice in the face of complexity and comorbidity: a case study of an adolescent with asperger’s syndrome, anxiety, depression, and chronic pain. J Child Adolesc Psychiatr Nurs.

[12] Bursch B, Ingman K, Vitti L, Hyman P, Zeltzer LK (2004). Chronic pain in individuals with previously undiagnosed autistic spectrum disorders. J Pain.

[13] Vorstman JAS, Parr JR, Moreno-De-Luca D, Anney RJL, Nurnberger JI, Hallmayer JF (2017). Autism genetics: opportunities and challenges for clinical translation. Nat Rev Genet.

[14] Han Q, Kim YH, Wang X, Liu D, Zhang Z-J, Bey AL (2016). SHANK3 deficiency impairs heat hyperalgesia and TRPV1 signaling in primary sensory neurons. Neuron.

[15] Braz J, Solorzano C, Wang X, Basbaum A (2014). Transmitting pain and itch messages: a contemporary view of the spinal cord circuits that generate gate control. Neuron.

[16] Nelson SB, Valakh V (2015). Excitatory/inhibitory balance and circuit homeostasis in autism spectrum disorders. Neuron.

[17] Sheng M, Kim E (2000). The Shank family of scaffold proteins. J Cell.

[18] Bourgeron T (2015). From the genetic architecture to synaptic plasticity in autism spectrum disorder. Nat Rev Neurosci.

[19] Schmeisser MJ, Ey E, Wegener S, Bockmann J, Stempel AV, Kuebler A (2012). Autistic-like behaviours and hyperactivity in mice lacking ProSAP1/Shank2. Nature.

[20] Won H, Lee H-R, Gee HY, Mah W, Kim J-I, Lee J (2012). Autistic-like social behaviour in Shank2-mutant mice improved by restoring NMDA receptor function. Nature.

[21] Yoon S-Y, Kwon S-G, Kim YH, Yeo J-H, Ko H-G, Roh D-H (2017). A critical role of spinal Shank2 proteins in NMDA-induced pain hypersensitivity. Mol Pain.

[22] Pappas AL, Bey AL, Wang X, Rossi M, Kim YH, Yan H (2017). Deficiency of Shank2 causes mania-like behavior that responds to mood stabilizers. JCI insight.

[23] Zeilhofer HU, Studler B, Arabadzisz D, Schweizer C, Ahmadi S, Layh B (2005). Glycinergic neurons expressing enhanced green fluorescent protein in bacterial artificial chromosome transgenic mice. J Comp Neurol.

[24] Hippenmeyer S, Vrieseling E, Sigrist M, Portmann T, Laengle C, Ladle DR (2005). A developmental switch in the response of DRG neurons to ETS transcription factor signaling. PLOS Biol.

[25] Vong L, Ye C, Yang Z, Choi B, Chua S, Lowell BB (2011). Leptin action on GABAergic neurons prevents obesity and reduces inhibitory tone to POMC neurons. Neuron.

[26] Kawaguchi Y, Cooper B, Gannon M, Ray M, MacDonald RJ, Wright CVE (2002). The role of the transcriptional regulator Ptf1a in converting intestinal to pancreatic progenitors. Nat Genet.

[27] Bechara A, Laumonnerie C, Vilain N, Kratochwil CF, Cankovic V, Maiorano NA (2015). Hoxa2 selects barrelette neuron identity and connectivity in the mouse somatosensory brainstem. Cell Rep.

[28] Hoshi M, Batourina E, Mendelsohn C, Jain S (2012). Novel mechanisms of early upper and lower urinary tract patterning regulated by RetY1015 docking tyrosine in mice. Development.

[29] Boeckers TM, Kreutz MR, Winter C, Zuschratter W, Smalla KH, Sanmarti-Vila L (1999). Proline-rich synapse-associated protein-1/cortactin binding protein 1 (ProSAP1/CortBP1) is a PDZ-domain protein highly enriched in the postsynaptic density. J Neurosci.

[30] Boeckers TM, Liedtke T, Spilker C, Dresbach T, Bockmann J, Kreutz MR (2005). C-terminal synaptic targeting elements for postsynaptic density proteins ProSAP1/Shank2 and ProSAP2/Shank3. J Neurochem.

[31] Roselli F, Livrea P, Almeida OFX (2011). CDK5 is essential for soluble amyloid β-induced degradation of GKAP and remodeling of the synaptic actin cytoskeleton. PLOS ONE.

[32] Roselli F, Hutzler P, Wegerich Y, Livrea P, Almeida OFX (2009). Disassembly of shank and homer synaptic clusters is driven by soluble b-amyloid 1–40 through divergent NMDAR-dependent signalling pathways. PLOS ONE.

[33] Li S, Olde Heuvel F, Rehman R, Aousji O, Froehlich A, Li Z (2023). Interleukin-13 and its receptor are synaptic proteins involved in plasticity and neuroprotection. Nat Commun.

[34] Saxena S, Roselli F, Singh K, Leptien K, Julien J-P, Gros-Louis F (2013). Neuroprotection through excitability and mTOR required in ALS motoneurons to delay disease and extend survival. Neuron.

[35] Li L, Rutlin M, Abraira VE, Cassidy C, Kus L, Gong S (2011). The functional organization of cutaneous low-threshold mechanosensory neurons. Cell.

[36] Ouali Alami N, Schurr C, Olde Heuvel F, Tang L, Li Q, Tasdogan A (2018). NF-κB activation in astrocytes drives a stage-specific beneficial neuroimmunological response in ALS. EMBO J.

[37] Chung K, Wallace J, Kim SY, Kalyanasundaram S, Andalman AS, Davidson TJ (2013). Structural and molecular interrogation of intact biological systems. Nature.

[38] Wang F, Flanagan J, Su N, Wang LC, Bui S, Nielson A (2012). RNAscope: a novel in situ RNA analysis platform for formalin-fixed, paraffin-embedded tissues. J Mol Diagn.

[39] Olde Heuvel F, Holl S, Chandrasekar A, Li Z, Wang Y, Rehman R (2019). STAT6 mediates the effect of ethanol on neuroinflammatory response in TBI. Brain Behav Immun.

[40] Osseforth C, Moffitt JR, Schermelleh L, Michaelis J (2014). Simultaneous dual-color 3D STED microscopy. Opt Express.

[41] Wang X, Zhang J, Eberhart D, Urban R, Meda K, Solorzano C (2013). Excitatory superficial dorsal horn interneurons are functionally heterogeneous and required for the full behavioral expression of Pain and Itch. Neuron.

[42] Lu J, Luo C, Bali KK, Xie R-G, Mains RE, Eipper BA (2015). A role for Kalirin-7 in nociceptive sensitization via activity-dependent modulation of spinal synapses. Nat Commun.

[43] Roome RB, Bourojeni FB, Mona B, Rastegar-Pouyani S, Blain R, Dumouchel A (2020). Phox2a defines a developmental origin of the anterolateral system in mice and humans. Cell Rep.

[44] Maricich SM, Morrison KM, Mathes EL, Brewer BM (2012). Rodents rely on Merkel cells for texture discrimination tasks. J Neurosci.

[45] Yalcin I, Charlet A, Freund-Mercier MJ, Barrot M, Poisbeau P (2009). Differentiating thermal allodynia and hyperalgesia using dynamic hot and cold plate in rodents. J Pain.

[46] Moqrich A, Hwang SW, Earley TJ, Petrus MJ, Murray AN, Spencer KSR (2005). Impaired thermosensation in mice lacking TRPV3, a heat and camphor sensor in the skin. Science.

[47] Chaplan SR, Bach FW, Pogrel JW, Chung JM, Yaksh TL (1994). Quantitative assessment of tactile allodynia in the rat paw. J Neurosci Methods.

[48] Tjølsen A, Berge OG, Hunskaar S, Rosland JH, Hole K (1992). The formalin test: an evaluation of the method. Pain.

[49] Zylka MJ, Rice FL, Anderson DJ (2005). Topographically distinct epidermal nociceptive circuits revealed by axonal tracers targeted to Mrgprd. Neuron.

[50] Witschi R, Punnakkal P, Paul J, Walczak JS, Cervero F, Fritschy JM (2011). Presynaptic alpha2-GABAA receptors in primary afferent depolarization and spinal pain control. J Neurosci.

[51] Szabo NE, Da Silva RV, Sotocinal SG, Zeilhofer HU, Mogil JS, Kania A (2015). Hoxb8 intersection defines a role for Lmx1b in excitatory dorsal horn neuron development, spinofugal connectivity, and nociception. J Neurosci.

[52] Eggeling C, Willig KI, Barrantes FJ (2013). STED microscopy of living cells—new frontiers in membrane and neurobiology. J Neurochem.

[53] Lim S, Naisbitt S, Yoon J, Hwang JI, Suh PG, Sheng M (1999). Characterization of the Shank family of synaptic proteins Multiple genes, alternative splicing, and differential expression in brain and development. J Biol Chem.

[54] Orefice LL, Mosko JR, Morency DT, Wells MF, Tasnim A, Mozeika SM (2019). Targeting peripheral somatosensory neurons to improve tactile-related phenotypes in ASD models. Cell.

[55] Woelfle S, Deshpande D, Feldengut S, Roselli F, Deisseroth K, Michaelis J, et al. CLARITY increases sensitivity and specificity of fluorescence immunostaining in long-term archived human brain tissue. bioRxiv. 2022;2022.04.27.489700. 10.1186/s12915-023-01582-6 PMC10207789 37221592

[56] Rebelo S, Reguenga C, Lopes C, Lima D (2010). Prrxl1 is required for the generation of a subset of nociceptive glutamatergic superficial spinal dorsal horn neurons. Dev Dyn.

[57] Hsu W, Mirando AJ, Yu HMI (2010). Manipulating gene activity in Wnt1-expressing precursors of neural epithelial and neural crest cells. Dev Dyn.

[58] Borromeo MD, Meredith DM, Castro DS, Chang JC, Tung KC, Guillemot F (2014). A transcription factor network specifying inhibitory versus excitatory neurons in the dorsal spinal cord. Development.

[59] Foster E, Wildner H, Tudeau L, Haueter S, Ralvenius WT, Jegen M (2015). Targeted ablation, silencing, and activation establish glycinergic dorsal horn neurons as key components of a spinal gate for pain and itch. Neuron.

[60] Alvarez FJ, Villalba RM, Zerda R, Schneider SP (2004). Vesicular glutamate transporters in the spinal cord, with special reference to sensory primary afferent synapses. J Comp Neurol.

[61] Bourane S, Duan B, Koch SC, Dalet A, Britz O, Garcia-Campmany L (2015). Gate control of mechanical itch by a subpopulation of spinal cord interneurons. Science.

[62] Ross SE, Mardinly AR, McCord AE, Zurawski J, Cohen S, Jung C (2010). Loss of inhibitory interneurons in the dorsal spinal cord and elevated itch in Bhlhb5 mutant mice. Neuron.

[63] Zeilhofer HU, Zeilhofer UB (2008). Spinal dis-inhibition in inflammatory pain. Neurosci Lett.

[64] Zeilhofer HU, Acuña MA, Gingras J, Yévenes GE (2018). Glycine receptors and glycine transporters: Targets for novel analgesics?. Cell Mol Life Sci.

[65] Lu Y, Dong H, Gao Y, Gong Y, Ren Y, Gu N (2013). A feed-forward spinal cord glycinergic neural circuit gates mechanical allodynia. J Clin Invest.

[66] Vuilleumier PH, Fritsche R, Schliessbach J, Schmitt B, Arendt-Nielsen L, Zeilhofer HU (2018). Mutations affecting glycinergic neurotransmission in hyperekplexia increase pain sensitivity. Brain.

[67] Zeilhofer HU, Neumann E, Munro G (2019). Spinal GABAA receptors for pain control: Back to the future?. Br J Anaesth.

[68] Boyle KA, Gradwell MA, Yasaka T, Dickie AC, Polgár E, Ganley RP (2019). Defining a spinal microcircuit that gates myelinated afferent input: implications for tactile allodynia. Cell Rep.

[69] Petitjean H, Pawlowski SA, Fraine SL, Sharif B, Hamad D, Fatima T (2015). Dorsal horn parvalbumin neurons are gate-keepers of touch-evoked pain after nerve injury. Cell Rep.

[70] Ralvenius WT, Benke D, Acuña MA, Rudolph U, Zeilhofer HU (2015). Analgesia and unwanted benzodiazepine effects in point-mutated mice expressing only one benzodiazepine-sensitive GABAA receptor subtype. Nat Commun.

[71] Ko HG, Oh SB, Zhuo M, Kaang BK (2016). Reduced acute nociception and chronic pain in Shank2^−/−^ mice. Mol Pain.

[72] Tsuda M, Koga K, Chen T, Zhuo M (2017). Neuronal and microglial mechanisms for neuropathic pain in the spinal dorsal horn and anterior cingulate cortex. J Neurochem.

[73] Häring M, Zeisel A, Hochgerner H, Rinwa P, Jakobsson JET, Lönnerberg P (2018). Neuronal atlas of the dorsal horn defines its architecture and links sensory input to transcriptional cell types. Nat Neurosci.

[74] Schlüter OM, Xu W, Malenka RC (2006). Alternative N-terminal domains of PSD-95 and SAP97 govern activity-dependent regulation of synaptic AMPA receptor function. Neuron.

[75] Favaro PD, Huang X, Hosang L, Stodieck S, Cui L, Liu Y-Z (2018). An opposing function of paralogs in balancing developmental synapse maturation. PLOS Biol.

[76] Shi R, Redman P, Ghose D, Hwang H, Liu Y, Ren X (2017). Shank proteins differentially regulate synaptic transmission. Eneuro..

[77] Monteiro P, Feng G (2017). SHANK proteins: roles at the synapse and in autism spectrum disorder. Nat Rev Neurosci.

[78] Chung C, Ha S, Kang H, Lee J, Um SM, Yan H (2019). Early correction of N-Methyl-D-aspartate receptor function improves autistic-like social behaviors in adult Shank2^−/−^ mice. Biol Psychiatry.

[79] Coderre TJ, Vaccarino AL, Melzack R (1990). Central nervous system plasticity in the tonic pain response to subcutaneous formalin injection. Brain Res.

[80] Ji RR, Baba H, Brenner GJ, Woolf CJ (1999). Nociceptive-specific activation of ERK in spinal neurons contributes to pain hypersensitivity. Nat Neurosci.

[81] Asante CO, Wallace VC, Dickenson AH (2009). Formalin-induced behavioural hypersensitivity and neuronal hyperexcitability are mediated by rapid protein synthesis at the spinal level. Mol Pain.

[82] Imlach WL, Bhola RF, Mohammadi SA, Christie MJ (2016). Glycinergic dysfunction in a subpopulation of dorsal horn interneurons in a rat model of neuropathic pain. Sci Rep.

[83] Orefice LL, Zimmerman AL, Chirila AM, Sleboda SJ, Head JP, Ginty DD (2016). Peripheral mechanosensory neuron dysfunction underlies tactile and behavioral deficits in mouse models of ASDs. Cell.

[84] Dawes JM, Weir GA, Middleton SJ, Patel R, Chisholm KI, Pettingill P (2018). Immune or genetic-mediated disruption of CASPR2 causes pain hypersensitivity due to enhanced primary afferent excitability. Neuron.

[85] Loomes R, Hull L, Mandy WPL (2017). What is the male-to-female ratio in autism spectrum disorder? A systematic review and meta-analysis. J Am Acad Child Adolesc Psychiatry.

[86] Dedek A, Xu J, Lorenzo LÉ, Godin AG, Kandegedara CM, Glavina G (2022). Sexual dimorphism in a neuronal mechanism of spinal hyperexcitability across rodent and human models of pathological pain. Brain.

[87] Mogil JS (2020). Qualitative sex differences in pain processing: emerging evidence of a biased literature. Nat Rev Neurosci.

