# Supplementary material for: Shank2 identifies a subset of glycinergic neurons involved in altered nociception in an autism model
Source: Mol Autism. 2023 Jun 14;14:21. doi: 10.1186/s13229-023-00552-7 (PMC10265811; doi:10.1186/s13229-023-00552-7)
Supplement: Supplementary file 1 — Additional file 1: Figure S1: Shank2 spinal cord localisation. Figure S2 Shank2 architecture and localisation. Figure S3 Loss of Shank2 immunoreactivity in cerebral cortex from Shank2−/− mice. Figure S4 CTB-488 injections reveal cutaneous afferents on Shank2high expressing neurons. Figure S5 No difference in neuronal architecture in the spinal cord of Shank2−/− mice. Figure S6 No difference in inhibitory synapses in the spinal cord of Shank2−/− mice. Figure S7 Increased number of cFos mRNA + interneurons in Laminae I upon Formalin injection. Table S1 Primary and secondary antibodies used for immunostainingFluorescent in Situ Hybridisationor for western blot. Table S2 Full details regarding human samples. Source data file 1 uncropped Western Blot of Shank2 in mouse tissue. [file 13229_2023_552_MOESM1_ESM.pdf]

Additional File 1

- Supplementary Figure 1
- Supplementary Figure 2
- Supplementary Figure 3
- Supplementary Figure 4
- Supplementary Figure 5
- Supplementary Figure 6
- Supplementary Figure 7
- Supplementary Table 1
- Supplementary Table 2
- Source data file 1

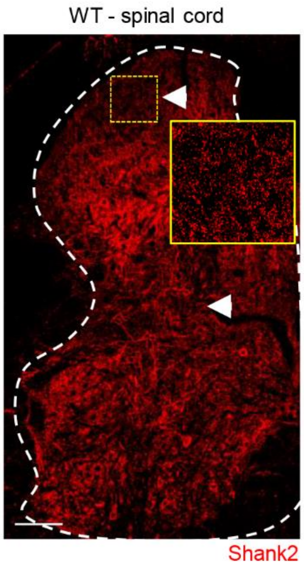

Supplementary Figure 1: Shank2 spinal cord localisation.

Immunofluorescence staining of WT spinal cord sections (unfiltered image from figure 2a) show differential expression level of Shank2 throughout the spinal cord, with a baseline level of Shank2 in laminae I, II and laminae VII (white arrows), a higher expression of Shank2 was observed in laminae III-V (Shank2^{high} cells) and in the ventral horn. N = 3. Scale bar 100µm.

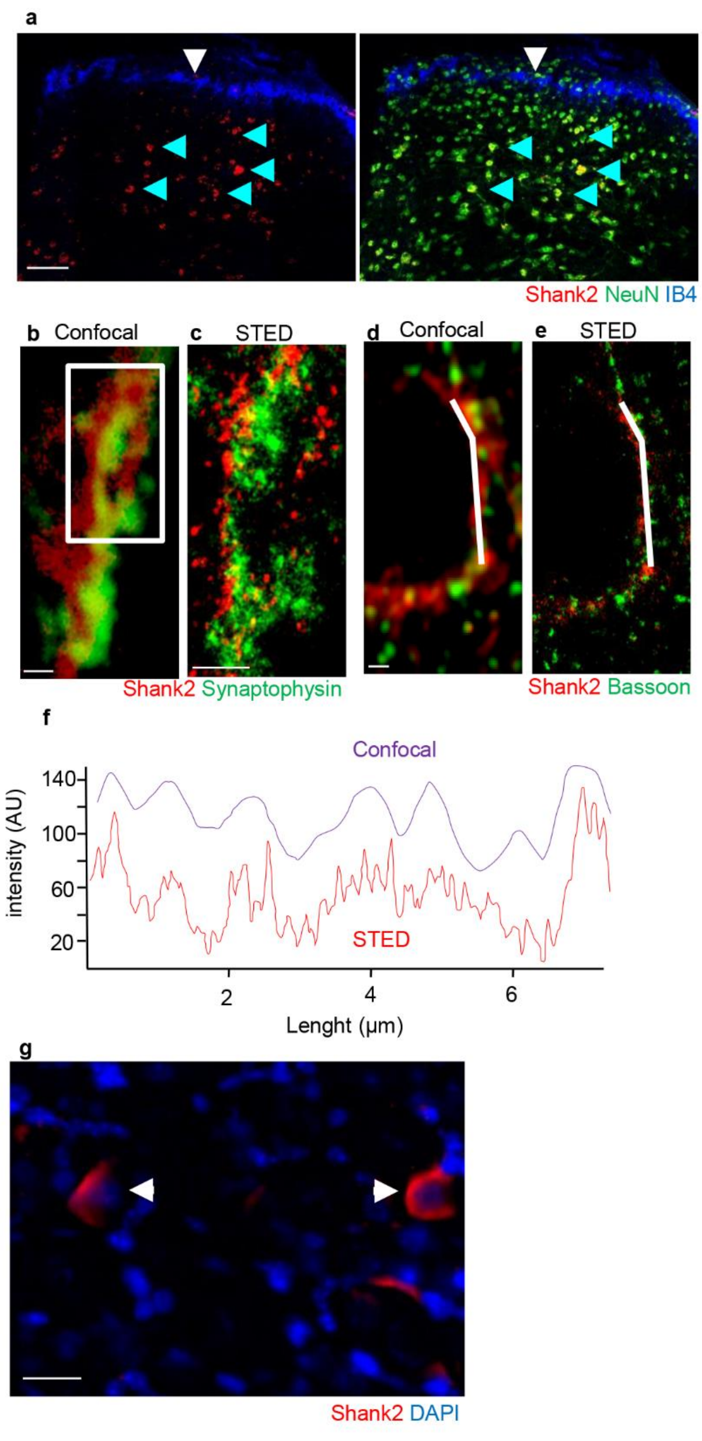

Supplementary Figure 2: Shank2 architecture and localisation.

(a) Confocal images of Shank2 together with IB4 identify Shank2^{high} neurons are located below laminae II (approx. 90%), with a small number of Shank2^{high} neurons in the IB4 ribbon. **(b)** Confocal images of Shank2 and Synaptophysin show a band of Shank2 colocalized with Synaptophysin; (N = 3) scale bar: 500nm. **(c)** STED images of Shank2 and Synaptophysin reveal the punctuated pattern of Shank2 and Synaptophysin; (N = 3) scale bar: 500nm. **(d)** Confocal images of Shank2 and Bassoon show a band of Shank2 colocalized with Bassoon puncta; (N = 3) scale bar: 500nm. **(e)** STED images of Shank2 and Bassoon reveal the punctuated pattern of Shank2 with Bassoon; (N = 3) scale bar: 500nm. **(f)** Graphical view of the fluorescence intensity of STED imaging in comparison to confocal microscopy. **(g)** Dorsal root ganglion staining with Shank2 revealed low shank2 intensity in only a few cells; scale bar: 10µm.

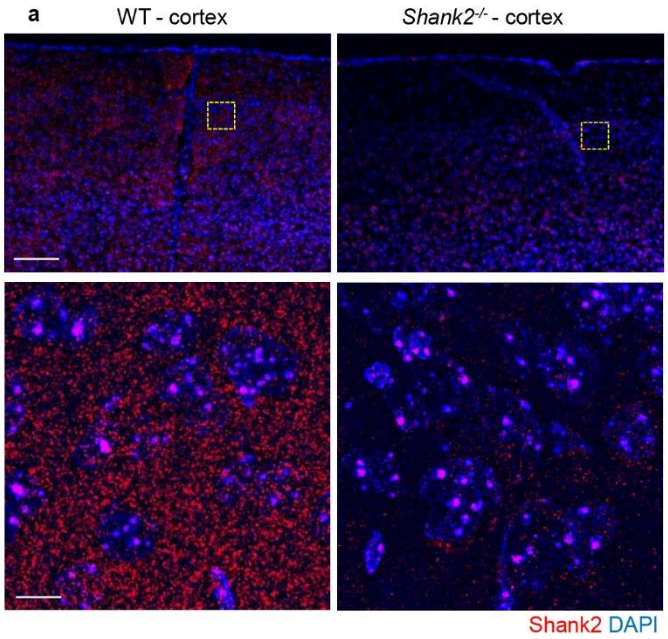

Supplementary Figure 3: Loss of Shank2 immunoreactivity in cerebral cortex from Shank2$^{-/-}$ mice.

(a) Shank2 immunofluorescence staining of WT and Shank2$^{-/-}$ cortical sections shows a homogeneous expression of Shank2 in WT and little to no expression in Shank2$^{-/-}$ sections. N = 3. Scale bar overview: 100µm; insert: 10µm.

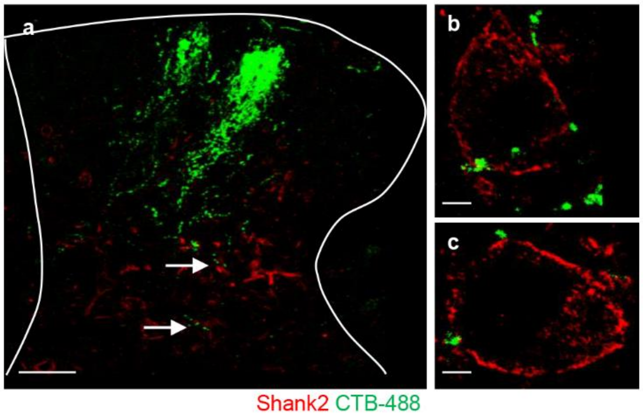

Supplementary Figure 4: CTB-488 injections reveal cutaneous afferents on Shank2^{high} expressing neurons.

(a-c) Detection of GFP positive buttons on Shank2 positive terminals after Cholera Toxin B subunit injection in the hairy skin to show cutaneous afferents on Shank2^{high} expressing neurons; (N = 3) scale bar; A: 50µm, scale bar B and C: 5µm.

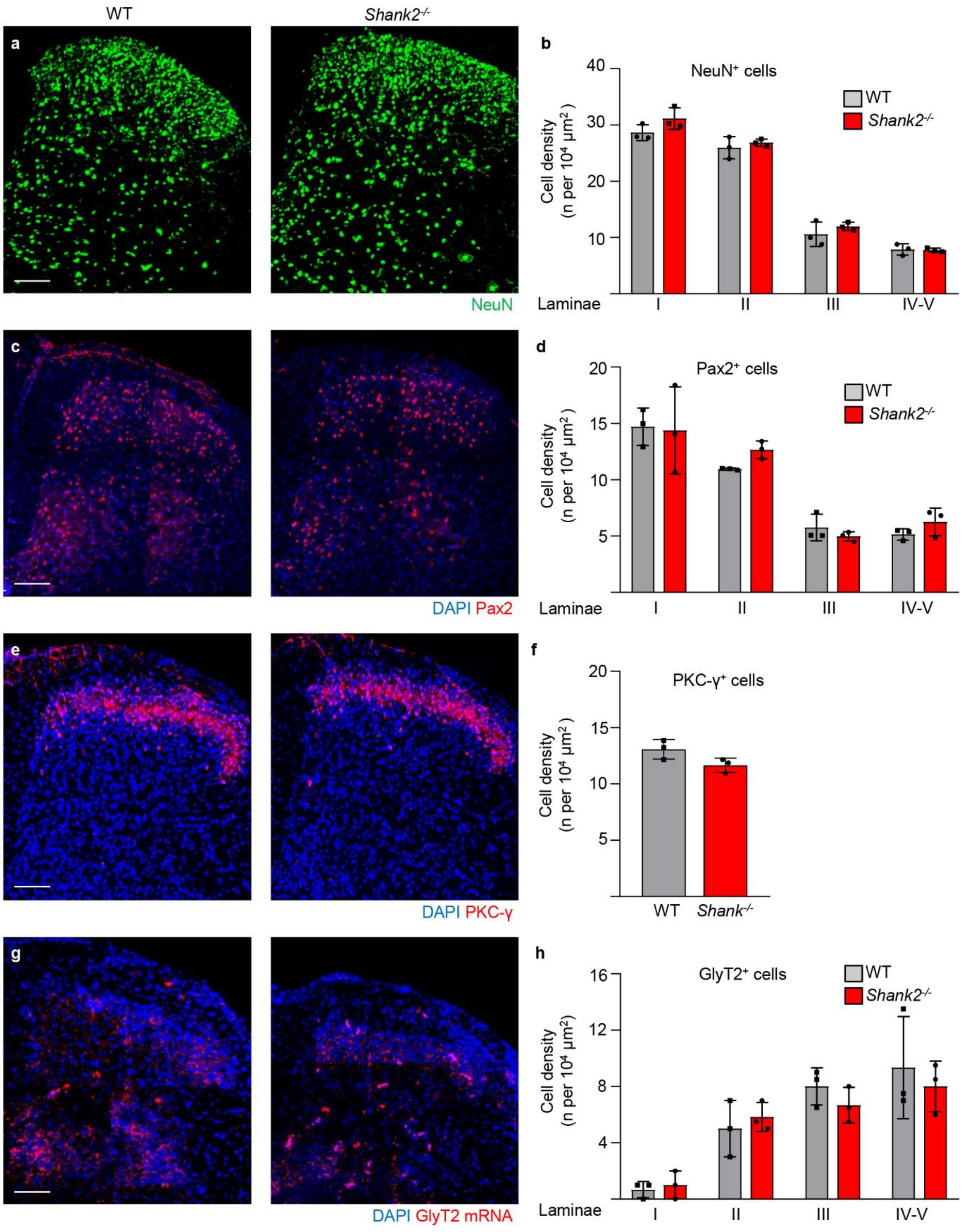
WT
Shank2-/-
a
b
NeuN+ cells
WT
Shank2-/-
Cell density
(n per 10⁴ µm²)
40
30
20
10
Laminae
I
II
III
IV-V
NeuN
c
d
Pax2+ cells
20
15
10
5
DAPI Pax2
e
f
PKC-γ+ cells
WT
Shank-/-
DAPI PKC-γ
g
h
GlyT2+ cells
16
12
8
4
DAPI GlyT2 mRNA

Supplementary Figure 5: No difference in neuronal architecture in the spinal cord of Shank2$^{-/-}$ mice.

(a, b) Neu-N staining in the spinal cord reveals no differences in Neuronal counts between the WT and Shank2$^{-/-}$ across Laminae (29.4±1.9 vs 30.3±2.3; 26.1±1.6 vs 25.5±2.7; 11.1±2.1 vs 12.0±0.6; 7.8±0.8 vs 7.9±0.4; per 10^4 µm^2; Laminae I-IV/V respectively; WT vs Shank2$^{-/-}$; p>0.05); (N = 3) scale bar: 100nm. **(c, d)** PAX2 staining in the spinal cord shows no differences in the density of inhibitory neurons between the WT and Shank2$^{-/-}$ (15.2±1.4 vs 15.0±3.2; 11.2±0.5 vs 12.2±0.7; 5.9±1.1 vs 4.7±0.7; 5.3±0.4 vs 5.7±1.0; per 10^4 µm^2; Laminae I-IV/V respectively; WT vs Shank2$^{-/-}$; p>0.05); (N = 3) scale bar: 100nm. **(e, f)** PKC-y staining in the spinal cord results in no difference in the number of excitatory cells between WT and Shank2$^{-/-}$ (13.2±1.5 vs 11.7±1.3; per 10^4 µm^2; WT vs Shank2$^{-/-}$; p>0.05); (N = 3) scale bar: 100nm. **(g, h)** single mRNA in situ hybridisation with GlyT2 in the spinal cord reveals no differences in the density of Glycinergic interneurons between WT and Shank2$^{-/-}$ (0.1±0.1 vs 0.1±0.2; 1.4±0.5 vs 1.5±0.4; 1.6±0.5 vs 1.7±0.4; 2.2±0.7 vs 2.1±0.5; per 10^4 µm^2; Laminae I-IV/V respectively; WT vs Shank2$^{-/-}$; p>0.05); (N = 3) scale bar: 100nm. Data shown as average±SD.

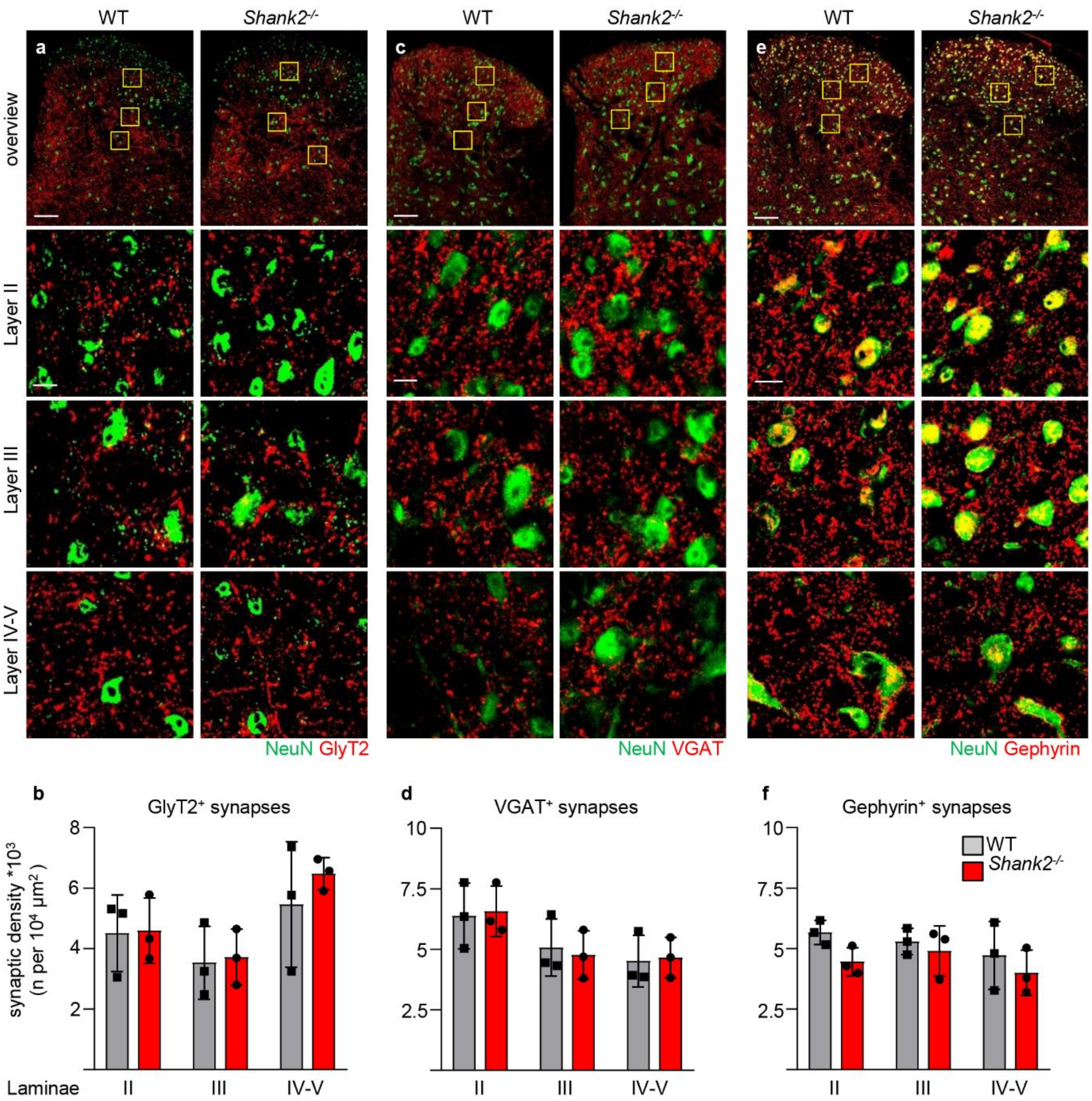

Supplementary Figure 6: No difference in inhibitory synapses in the spinal cord of Shank2$^{-/-}$ mice.

(a, b) GlyT2 staining in the spinal cord reveals no differences in the GlyT2 synaptic density between WT and Shank2$^{-/-}$ across all laminae (4.5±1.6 vs 4.6±1.4; 3.5±1.7 vs 3.7±1.1; 5.5±2.1 vs 6.5±8.8 $*10^3$; per 10^4 μm^2; Laminae II, III and IV respectively; WT vs Shank2$^{-/-}$; p>0.05); (N = 3). **(c, d)** VGAT staining in the spinal cord reveals no differences in the VGAT synaptic density between WT and Shank2$^{-/-}$ across all laminae

(6.3±1.9 vs 6.5±1.5; 4.9±1.6 vs 4.7±1.4; 4.4±1.4 vs 4.6±1.0 $*10^3$; per 10^4 μm^2; Laminae II, III and IV respectively; WT vs Shank2$^{-/-}$; p>0.05); (N = 3). **(e, f)** Gephyrin staining in the spinal cord reveals no differences in the Gephyrin synaptic density between WT and Shank2$^{-/-}$ across all laminae (5.7±1.0 vs 4.5±1.6; 5.3±1.1 vs 4.9±1.8; 4.7±1.8 vs 4.0±1.5 $*10^3$; per 10^4 μm^2; Laminae II, III and IV respectively; WT vs Shank2$^{-/-}$; p>0.05); (N = 3). Scale bar overview: 100µm, scale bar insert: 10µm. Data shown as average±SD.

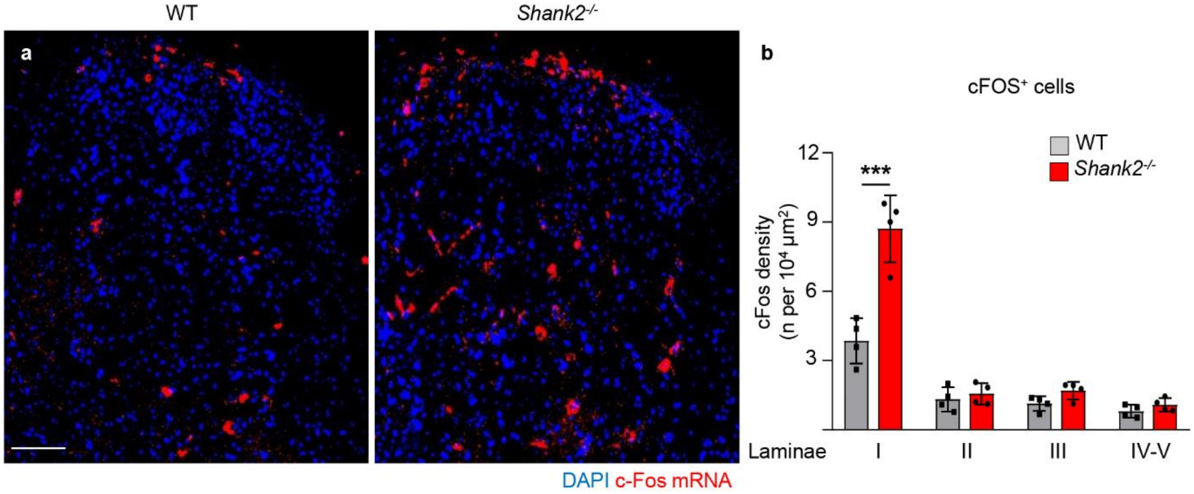

Supplementary Figure 7: Increased number of cFos mRNA+ interneurons in Laminae I upon Formalin injection

(a, b) Single mRNA detection show a strong increase in c-fos expressing cells in laminae I ($p<0.001$), however this effect disappears in Laminae II-IV/V (1.5±0.6 vs 1.6±0.5; 1.2±0.3 vs 1.7±0.4; 0.8±0.2 vs 1.1±0,3; per 10^4 μm^2; Laminae II-IV/V respectively; WT vs Shank2$^{-/-}$; $p>0.05$) upon formalin injection; (N = 4) scale bar A: 100µm. Data shown as average±SD. *** $p < 0.001$.

Antigen (primary antibody)	Catalogue number	Company	Dilution
Chicken anti GFP	Ab13970	Abcam	1:1000 (IF)
Rabbit anti RFP	600-401-379	Rockland	1:500 (IF)
Goat anti Parvalbumin	PVG-214	Swant	1:2000 (IF)
Mouse anti Gephyrin	147011	Synaptic Systems	1:500 (IF)
Goat anti Glycine Transporter 2	SC-16705	Santa Cruz	1:200 (IF)
Rabbit anti Glycine Transporter 2	272003	Synaptic Systems	1:200 (IF)
Mouse anti NeuN	MAB377	Millipore	1:100 (IF)
Guinea pig anti vGlut1	135304	Synaptic Systems	1:500 (IF), 1:200 (RNAscope, clearing)
Guinea pig anti vGlut2	135404	Synaptic Systems	1:500 (IF), 1:200 (RNAscope)
Guinea pig anti VGAT	131004	Synaptic Systems	1:500 (IF)
Rabbit anti c-fos	SC-52	Santa Cruz	1:500 (IF)
Mouse anti Bassoon	ADI-VAM-PS003-D	Enzo Life Sciences	1:500 (IF, STED)
Mouse anti Synaptophysin	MAB5258	Millipore	1:1000 (IF, STED)
Rabbit anti Pax-2	PRB-2768	Covance	1:500 (IF)
Rabbit anti PKC-gamma	Ab71558	Abcam	1:200 (IF)
Rabbit anti Shank2	Homemade	SA5192	1:500 (IF, STED, WB), 1:200 (Clearing)
Rabbit anti Homer 1b/c	160022	Synaptic Systems	1:200 (RNAscope)
Mouse anti GluN1	114011	Synaptic Systems	1:200 (RNAscope)
Mouse anti Neurofilament Marker	SMI-311R	Biolegend	1:300 (IF)
Anti Gs-IB4 Alexa Fluor 488 conjugated	121411	Invitrogen	1:1000 (IF)
Chicken anti MAP2	CPCA-MAP2	Encor	1:200

Antigen (secondary antibody)	Catalogue number	Company	Dilution
Donkey anti mouse 488	A21202	Invitrogen	1:500
Donkey anti mouse 568	A10037	Invitrogen	1:500
Donkey anti rabbit 488	A21206	Invitrogen	1:500
Donkey anti rabbit 568	A10042	Invitrogen	1:500
Donkey anti rabbit 647	A31573	Invitrogen	1:500
Donkey anti chicken 488	SAB4600031	Sigma	1:500
Donkey anti goat 568	A11057	Invitrogen	1:500
Donkey anti goat 633	A21082	Invitrogen	1:500
Donkey anti guinea pig 568	20377	Biotium	1:500
Goat anti mouse 594 (Atto)	76085-1ML-F	Sigma-Aldrich	1:1000 (STED)
Goat anti rabbit 647 (Atto)	40839-1ML-F	Sigma-Aldrich	1:1000 (STED)
Goat anti guinea pig 633	A21105	Invitrogen	1:500

Supplementary Table 1: Primary and secondary antibodies used for immunostaining (IF) Fluorescent in Situ Hybridisation (RNAscope) or for western blot (WB).

Lab ID Nr.	Gender	Age
**34**	Female	69
**35**	Male	75
**39**	Female	84
**41**	Female	85
**44**	Male	94

Supplementary Table 2: Full details regarding human samples.

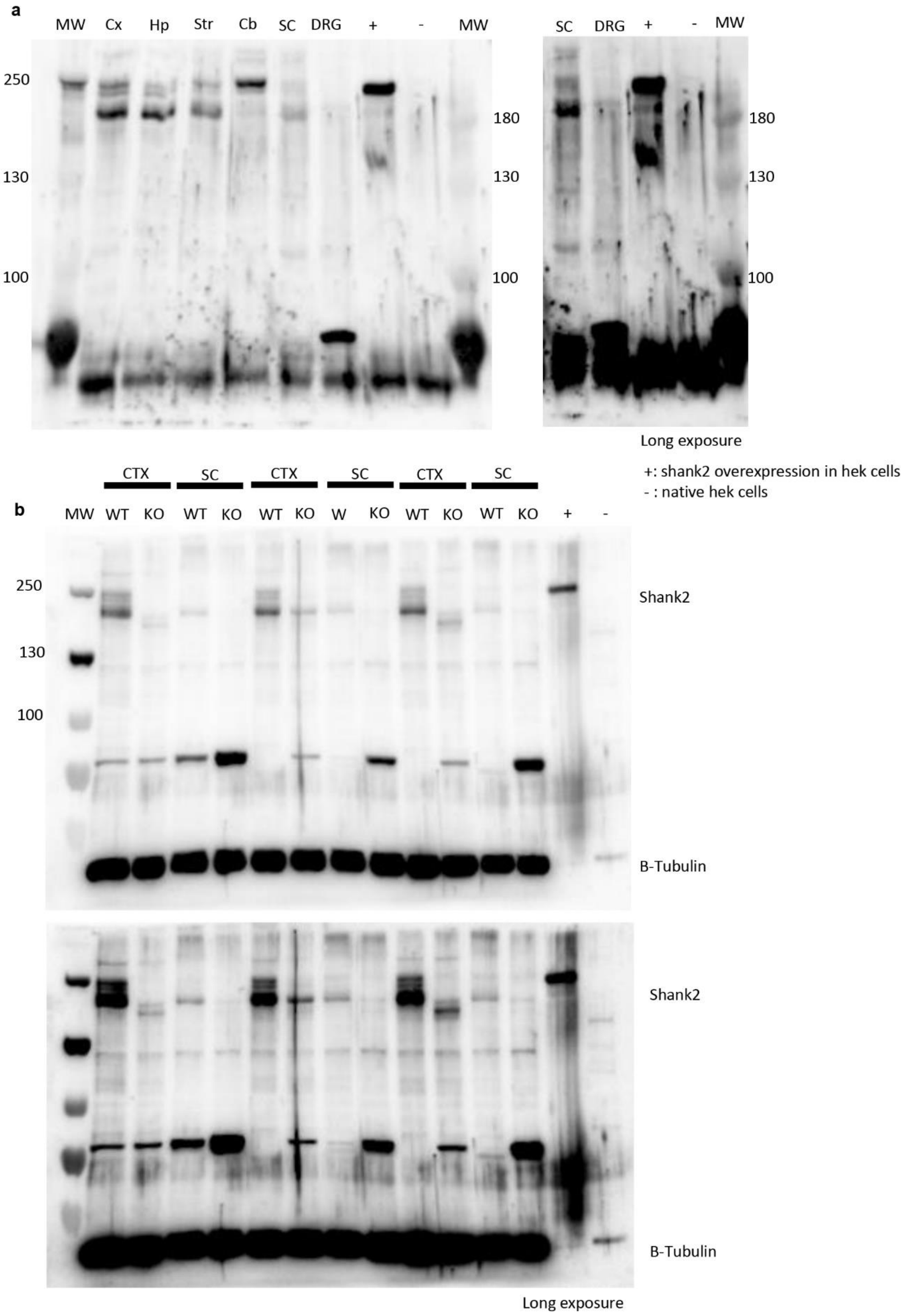
a
MW Cx Hp Str Cb SC DRG + - MW
250
130
100
180
130
100
SC DRG + - MW
180
130
100
Long exposure
+: shank2 overexpression in hek cells
- : native hek cells
b
CTX SC CTX SC CTX SC
MW WT KO WT KO WT KO W KO WT KO WT KO + -
250
130
100
Shank2
B-Tubulin
Shank2
B-Tubulin
Long exposure

Source data file 1: uncropped Western Blot of Shank2 in mouse tissue

(a) Shank2 western blot (WB) on mouse tissue samples of various central nervous system (CNS) regions; Cortex (Cx), Hippocampus (Hp), Striatum (Str); Cerebellum (Cb), Spinal Cord (SC), Dorsal Root Ganglia (DRG), positive control (+) and negative control (-). **(b)** Shank2 WB on cortex and spinal cord tissue of WT and Shank2$^{-/-}$ (KO) mice.
